# Zwitterionic Ionogels Resolving the Trade-Off Between Mechanical Strength and Autonomous Self-Healing for Iontronics

**DOI:** 10.1007/s40820-026-02318-1

**Published:** 2026-07-30

**Authors:** Zhengyang Kong, Ji Hong Kim, Jonghwi Kim, Woojin Lee, Hayoung Oh, Wu Bin Ying, Joo Sung Kim, Seonghwan Yun, So Young Kim, Do Hwan Kim

**Affiliations:** 1 Department of Chemical Engineering, Hanyang University, Seoul, 04763 Republic of Korea; 2 School of Electrical Engineering (EE), Korea Advanced Institute of Science and Technology (KAIST), Daejeon, 34141 Republic of Korea; 3 Present Address: Thin-Film Device Laboratory and Center for Emergent Matter Science (CEMS), RIKEN, 2-1 Hirosawa, Wako, Saitama 351-0198 Japan; 4 Institute of Nano Science and Technology, Hanyang University, Seoul, 04763 Republic of Korea

**Keywords:** Zwitterionic ionogels, High strength, Autonomous self-healing, Self-sustaining iontronics

## Abstract

Simultaneously achieving mechanical robustness and autonomous self-healing in ionogels remains a fundamental challenge for durable, skin-like electronics. Conventional approaches often improve mechanical strength by introducing rigid or densely cross-linked polymer networks, but such strategies inevitably restrict polymer chain mobility and hinder dynamic bond reconfiguration required for healing. Here, a zwitterionic side-chain engineered tough ionogel (ZESTI) is developed to overcome this trade-off through molecular-level design. Hydrophilic zwitterions are covalently grafted onto a hydrophobic polyurethane backbone to preferentially interact with the ionic liquid through ion–dipole interactions and thereby regulate its distribution. This architecture simultaneously facilitates dipole–dipole interactions for mechanical reinforcement and ion–dipole coordination for efficient self-healing under ambient conditions. As a result, ZESTI exhibits an exceptional combination of tensile strength (10.40 MPa), stretchability (1606%), toughness (56.03 MJ m^−3^), and ambient self-healing efficiency exceeding 83%, while maintaining high ionic conductivity via enhanced ion hopping. When constructed as a self-reporting packaging interface, ZESTI provides stable protection and state perception under sharp contact and restores signal output after mechanical damage through self-healing. This work offers a generalizable design strategy that reconciles mechanical toughness with dynamic functionality in ionogels, establishing a general design paradigm for next-generation self-sustaining iontronic devices.

**Supplementary Information:**

The online version contains supplementary material available at 10.1007/s40820-026-02318-1.

## Highlights

- Zwitterionic side-chain engineered tough ionogel (ZESTI) resolves the trade-off between mechanical toughness and autonomous self-healing through zwitterionic side-chain engineering.
- Synergistic dynamic ion–dipole and dipole–dipole interactions enable robust mechanical reinforcement, efficient ion transport, and room-temperature self-healing.
- ZESTI-based iontronic devices exhibit reliable sensing performance and functional recovery after mechanical damage.

**Supplementary Information:**

The online version contains supplementary material available at 10.1007/s40820-026-02318-1.

## Graphical abstract

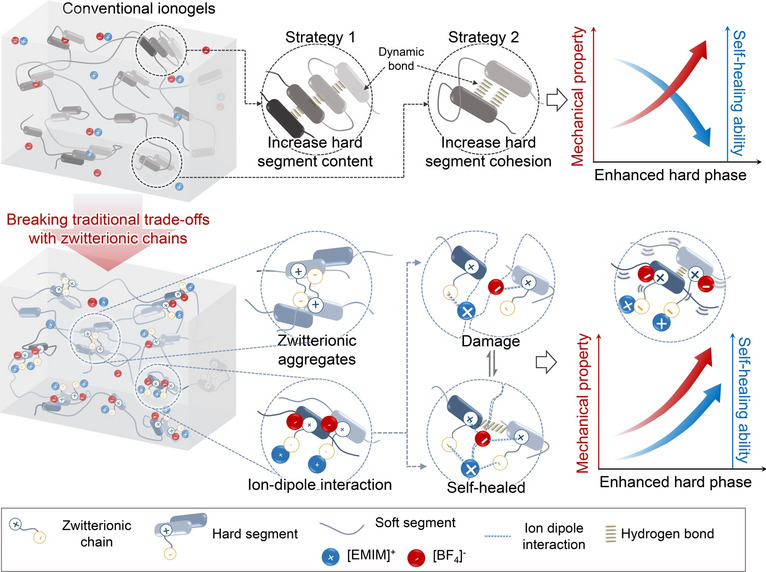

## Introduction

Flexible and wearable electronic skins are poised to transform applications into flexible electronics [1, 2], intelligent packaging [3, 4], soft robotics [5–7], and physically embodied artificial intelligence [8, 9]. A key prerequisite for these technologies is the development of materials that are not only intrinsically conductive but also mechanically robust and autonomously self-healing [10, 11]. These combined attributes are essential for ensuring long-term durability, enabling devices to withstand continuous mechanical deformation and recover from physical damage without external intervention. Ionogels, polymer networks swollen with ionic liquids, have emerged as promising candidates for such platforms due to their inherent ionic conductivity and mechanical flexibility [12]. However, achieving both high mechanical strength and efficient self-healing within a single ionogel remains a significant challenge [13, 14]. Conventional strategies often rely on rigid or densely cross-linked polymer networks to improve mechanical integrity [15–17]. While such networks enhance structural stability, they inevitably restrict polymer chain mobility, limiting the dynamic bond reconfiguration required for autonomous healing. Consequently, many mechanically strong ionogels demand external stimuli to initiate healing, which undermines their practicality in wearable applications. This long-standing trade-off between mechanical toughness and autonomous reparability continues to constrain the design space for truly sustainable iontronic materials.

To address this challenge, recent efforts have focused on engineering microphase-separated or hierarchically organized ionogel networks that couple mechanically reinforcing domains with dynamic soft phases within a single matrix [12, 18, 19]. Recent breakthroughs based on synergistic entanglement and multiphase interlocking architectures have further expanded this design space by improving mechanical robustness, deformation adaptability, and energy dissipation in high-performance ionogels [20–22]. These architectures highlight the importance of spatially organized networks for balancing strength and flexibility. However, mechanical reinforcement alone does not fully address the requirements of self-sustaining iontronic materials, because autonomous repair also requires reversible interactions, polymer chain reconfigurability, and efficient ion transport after damage. Polyurethanes (PUs) are widely recognized for their ability to form well-defined microphase-separated morphologies, owing to strong polarity contrast between their hard and soft segments [23, 24]. Physical cross-linking within PU hard domains, mediated by hydrogen bonding and dipole–dipole interactions, provides excellent mechanical strength and energy dissipation. Incorporating dynamic bonding motifs into PU networks has further enabled self-healing through reversible bond exchange. However, integrating ionic liquids (ILs) into PU-based ionogels remains inherently difficult, as ILs can disrupt hard-segment self-assembly, diminishing phase separation and undermining both mechanical performance and healing functionality.

Here, we report a zwitterion-enabled molecular engineering strategy that simultaneously imparts tough and spontaneous self-healing by leveraging reversible ion–dipole interactions in the network. Specifically, hydrophilic zwitterions are covalently grafted onto a hydrophobic polyurethane backbone, forming well-defined phase-separated domains. The zwitterionic blocks preferentially interact with [EMIM]^+^[BF_4_]^−^ through ion–dipole interactions, thereby preserving the physical cross-linking points within the hard segments while maintaining uniform IL distribution. Compared with typical zwitterionic ionogels in which zwitterionic groups mainly serve as ion-coordination or ion-transport sites, our design uses zwitterionic side chains to regulate the microphase-separated polyurethane architecture itself. This dual role enables the same molecular motif to preserve hard-segment cohesion, regulate the distribution of the ionic liquid, and provide reversible ion–dipole interactions for autonomous healing and efficient ion transport. Simultaneously, dipole–dipole interactions between zwitterionic moieties promote partial hard-domain aggregation, synergistically reinforcing the network. This design yields a zwitterionic side-chain engineered tough ionogel (ZESTI) that simultaneously achieves high tensile strength, large elongation, outstanding toughness, and robust Young’s modulus. Beyond mechanical enhancement, the zwitterionic motifs promote dynamic ion–dipole interactions with the IL, enabling autonomous self-healing at ambient temperature while suppressing plasticization. Additionally, these motifs facilitate efficient ion-hopping mechanisms, leading to enhanced ionic conductivity compared to conventional self-healing gels. Leveraging this multifunctional architecture, we employed the ZESTI as a multifunctional encapsulation platform while sustaining electrical and mechanical integrity under repeated deformation. Intrinsic self-healing nature enables rapid recovery of both mechanical toughness and ionic conductivity after damage, allowing reliable performance without external stimuli or resource-intensive repair processes. The combination of mechanical resilience, spontaneous healing, and efficient ion transport positions ZESTI as a generalizable and scalable material system for next-generation wearable iontronic technologies.

## Experimental Section

### Materials

Anhydrous tetrahydrofuran (THF, 99.5%), anhydrous *N*,*N*-dimethylformamide (DMF, 99.8%), isophorone diisocyanate (IPDI, 99%), poly(tetramethylene ether glycol) (PTMG, *M*_*n*_  ≈  2900 g mol^−1^), *N*-methyldiethanolamine (MDEA, 99%), 1,3-propanesultone (PS, 98%), dibutyltin dilaurate (DBTDL, 98%), and 1-ethyl-3-methylimidazolium tetrafluoroborate ([EMIM]^+^[BF_4_]^–^, 98%) were purchased from Sigma-Aldrich (South Korea) and used without further purification.

### Synthesis of PMPU

In a glove box filled with 99.999% N₂, PTMG (10.00 g), chain extender MDEA (2.50 g), catalyst DBTDL (0.25 g), and anhydrous THF (40 g) were added into a three-neck reactor equipped with a mechanical stirrer. After the reactants were completely dissolved at 45 °C, IPDI (5.78 g) was added using a vacuum syringe. The reaction mixture was heated at 70 °C for 6 h. The target product was washed several times with deionized water and then dried in a vacuum oven at 60 °C for 24 h to obtain PMPU (Scheme S1 and Fig. S1).

### Synthesis of Zwitterionic Side-Chain Engineered Thermoplastic Polyurethane (ZESTPU)

In a glove box filled with 99.999% N₂, PMPU (10.00 g), PS (2.10 g, molar ratio of PS/MDEA = 1:1.5), and DMF (40 g) were added into a round-bottom flask. The reaction was heated at 70 °C for 24 h. After the reaction, the solution was cast into a PTFE mold and placed on a heating plate for drying. Following solvent evaporation, the target product was further dried in a vacuum oven at 60 °C for 24 h to obtain ZESTPU (Scheme S2 and Fig. S1).

### Preparation of i-PMPU and ZESTI

ZESTI was prepared by dissolving ZESTPU and 30 wt% [EMIM]^+^[BF_4_]^–^ ionic liquid (IL) in DMF, while stirring at room temperature for 8 h. The solution was then poured into a square PTFE mold and annealed for 48–72 h at 60 °C under optimized conditions (initially starting at 40 °C with temperature intervals of 10 °C h^−1^). Notably, in this study, the weight percentage of IL represents the weight ratio of IL to IL + PU. Hence, ZESTI@10% was composed of 10 wt% IL + ZESTPU. Likewise, i-PMPU (used as reference) was prepared by combining PMPU and IL following the same procedure as described above.

### Material Characterization and Self-Healing Tests

Universal testing machine (UTM, Instron 5567) equipment was utilized to investigate the mechanical properties at room temperature. The strain rate was controlled at 10 mm min^–1^ using dumbbell-shaped splines with dimensions of 63.5 mm (length), 3 mm (width), and ~ 0.2 mm (thickness). X-ray photoelectron spectroscopy (XPS) measurements were performed using a K-Alpha Plus spectrometer (Thermo Scientific, USA) with a monochromatic Al K*α* X-ray source (*hν* = 1486.6 eV). X-ray diffraction analysis was performed using a Bruker D8 Advance diffractometer with Cu-Kα radiation (*λ* = 1.54 Å). Diffraction scans were collected over a 2θ range of 5°–50°, with a step time of 0.6 s. Attenuated total reflection-Fourier transform infrared (ATR-FTIR) spectroscopy spectra were obtained using a Bruker Optics GmbH (ZnSe instrument, Germany) spectrometer in the absorption mode. AFM phase images were acquired using Park Systems NX10 atomic force microscope operating in tapping mode. Small-angle X-ray scattering (SAXS) measurements were performed using a multipurpose X-ray diffractometer (Empyrean, PANanalytical, The Netherlands) equipped with the ScatterX^78^ stage and a Cu-Kα radiation source (*λ* = 1.54 Å). Rheological properties were tested on a rotational rheometer (Anton Paar, MCR102e) using a parallel plate geometry (diameter = 25 mm). Oscillatory amplitude sweep tests were performed at a constant angular frequency of 10 rad s^−1^ with strain ranging from 0.1 to 500%. Oscillatory time sweep tests were conducted at a constant strain of 1% and angular frequency of 10 rad s^−1^. Stress relaxation experiments were carried out by applying a constant strain (1%) and recording the decay of stress over time. For scratch self-healing experiments, a scratch recovery tests were performed under a real-time optical microscope (Olympus/BX 51TF Instec H601, Japan) for various time periods at ambient condition (20%–40% relative humidity). Elemental analysis was performed using an elemental analyzer (EA, FLASH SMART, Thermo Fisher Scientific, USA).

The self-healing efficiency was calculated based on the recovery of toughness, which was obtained by integrating the area under the stress–strain curve until fracture. After damage, the samples were allowed to heal at room temperature for 24 h. The self-healing efficiency was calculated using the following equation:

$${\text{Self{-}healing}}\;{\text{efficiency }} = { }\frac{{{\mathrm{Toughness}}_{{{\mathrm{healed}}}} }}{{{\mathrm{Toughness}}_{{{\mathrm{original}}}} }} \times 100\%$$

where Toughness_original_ and Toughness_healed_ represent the toughness of the original and healed samples, respectively. All mechanical tests were performed using three independent specimens.

### Electrical Characterization

Electrochemical impedance spectroscopy (EIS) data were collected at room temperature using an electrochemical analyzer PGSTAT302N (Metrohm Autolab) in a 0.1 Hz ~ 1 MHz frequency range with a 10 mV alternating current (AC) signal. The obtained impedance spectra were fitted using appropriate equivalent circuit models built in NOVA software (Metrohm Autolab), enabling the evaluation of the bulk resistance (R_b_) of the devices. The impedance spectra were fitted using an equivalent circuit composed of a series resistance *R*_s_, a bulk ionic resistance *R*_b_, and constant phase elements representing non-ideal bulk and electrode polarization. The ionic conductivity was calculated using *σ* = l/(*R*_b_A), where l is the film thickness, *R*_b_ is the fitted bulk resistance, and A is the electrode area.

### Device Demonstration System

To evaluate the potential application of high-performance ZESTI in flexible electronic devices, ZESTI-based self-reporting packaging interface and strain sensor devices were designed and fabricated for functional demonstration. In the device architecture, soft copper tape electrodes were integrated at both ends on the same side of the ZESTI to ensure mechanical compliance and stable electrical contact. For electrical performance assessment, the device was connected to a high-precision LCR meter (Agilent Keysight Technologies, E4980A) using silver wires, and systematic electrical characterizations, such as resistance response, were subsequently conducted.

## Results and Discussion

### Design and Development of High-Performance Ionogels via Zwitterionic Side-Chain Engineering

**Fig. 1:**
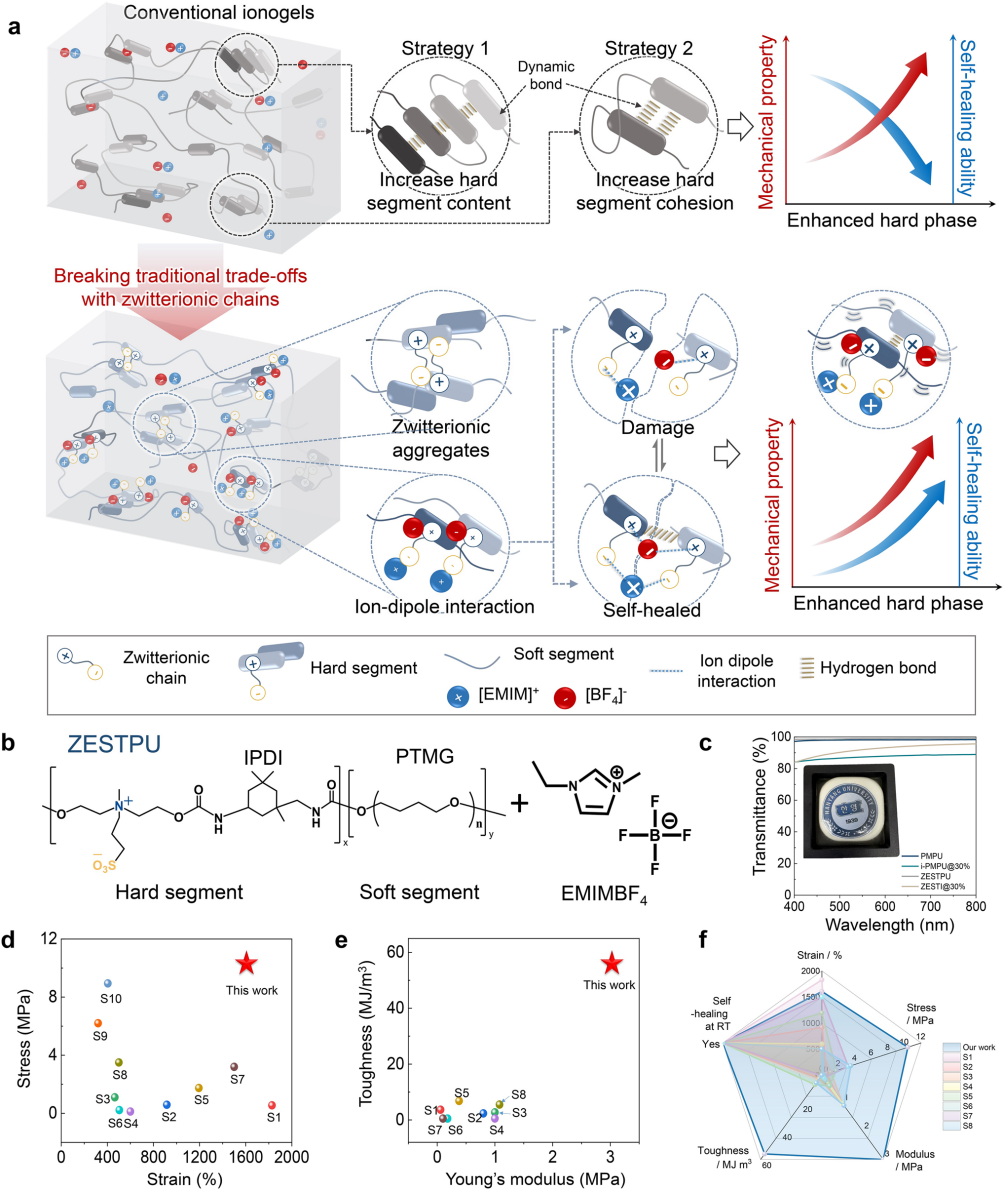
**a** Schematic illustration of traditional ionogels and ZESTI featuring aggregated zwitterionic chains, ion-transport channels, and dynamic interactions. **b** Representation of the design chemistry of ZESTPU and ionic liquid. **c** UV–Vis transmittance spectra of PMPU, i-PMPU, ZESTPU, and ZESTI. **d** Comparison of stress and strain performance between ZESTI and previously reported self-healing ionogels. **e** Comparison of Young’s modulus and toughness between ZESTI and previously reported self-healing ionogels. **f** Radar chart compares that the mechanical and self-healing properties of ZESTI with those of previously reported self-healing ionogels

We propose a molecular design strategy to simultaneously enhance mechanical strength, ambient self-healing, and ionic conductivity in ZESTI systems. This approach leverages the hydrophilicity, strong dipole moments, and anti-polyelectrolyte behavior of sulfonate-based zwitterionic side chains. By incorporating these hydrophilic moieties into a hydrophobic polyurethane backbone, we create a heterogeneous matrix that balances chain mobility and ionic interactions. The addition of hydrophilic ionic liquids further promotes dynamic ion transport, contributing to the material’s multifunctional performance. A schematic overview of the ZESTI design is provided in Fig. 1a. To realize this concept, a ZESTPU was synthesized by introducing zwitterionic chains into the hard segments of the polyurethane (PMPU) synthesized from IPDI, PTMG, and MDEA, and the detailed synthetic route is presented in Schemes S1 and S2. Ionogels, the incorporated zwitterionic side chains, possessing a high dipole moment, significantly enhance the cohesive forces between hard segments through strong dipole–dipole interactions, promoting the aggregation of adjacent hard phases [25, 26]. The resulting enhancement in microphase separation further increases the enrichment and stability of hard phases, enabling them to act as more effective dynamic physical cross-linking points and energy-dissipating units. During tensile deformation, these reinforced hard phases can dissipate mechanical energy through the dissociation and reformation of reversible noncovalent associations, thereby improving the strength and toughness of ZESTI.

To further prevent the disruption of the hydrophobic hard-segment stack, a hydrophilic ionic liquid, [EMIM]^+^[BF_4_]^−^, was selected due to its good compatibility with the zwitterionic chain. The sulfonate-type zwitterionic side chains exhibit a preferential ion–dipole interaction with hydrophilic ionic liquids enabled by their high dipole moment and anti-polyelectrolyte behavior [27]. These dynamic interactions promote effective mechanical energy dissipation under deformation, thereby enhancing mechanical performance [26]. Under an applied electric field, the zwitterionic side chains also tend to align along the field direction, forming efficient ion conduction pathways within the ionogel network [28, 29]. Additionally, the reversible nature of the ion–dipole interactions allows noncovalent bonds to repeatedly dissociate and reform, enabling ZESTI to self-heal efficiently at room temperature. We incorporated an ionic liquid into PMPU- and ZESTPU-based matrix (i-PMPU and ZESTI, respectively) to investigate the influence of zwitterionic side chains on the ionic liquid behavior within the ionogel network. Figure 1b shows the chemical structures of the ZESTPU and ionic liquid. As shown in Fig. 1c, all four samples exhibit excellent optical transparency, with transmittance exceeding 80% across the visible range (400–800 nm), suggesting that the ionogel networks remain optically homogeneous on the visible-light length scale. Notably, ZESTPU and PMPU show comparable transmittance values of approximately 98% and 99% at 550 nm, respectively, indicating that the introduction of zwitterionic side chains has a limited effect on the optical transparency of the polyurethane matrix. This result suggests that the zwitterionic component mainly modulates the weak microphase-separated structure without inducing large phase-separated domains or strong light scattering, which is advantageous for potential applications in flexible displays and wearable electronics.

Importantly, enabled by the zwitterionic side-chain engineering strategy, ZESTI (IL 30 wt%) simultaneously achieves outstanding mechanical strength, ambient self-healing capability, and high ionic conductivity. Compared with previously reported self-healing ionogels, ZESTI demonstrates superior performance in multiple mechanical parameters, including tensile strength, elongation at break, Young’s modulus, and toughness, achieving a comprehensive enhancement in mechanical properties, as shown in Fig. 1d, e. This comprehensive performance improvement highlights the effectiveness and advancement of the zwitterionic side-chain engineering strategy in constructing high-performance multifunctional ionogels. To provide a comprehensive assessment, a radar chart was constructed (Fig. 1f), in which ZESTI exhibits the largest integrated performance area among reported ionogels. We further summarized the key mechanical and self-healing properties of ZESTI and previously reported self-healing ionogels in Table S1, with the corresponding references listed in Table S2, in relation to the comparison shown in Fig. 1d–f. This result underscores its uniquely balanced combination of mechanical robustness and intrinsic self-healing, attributed to synergistic structural engineering at the molecular level.

### Structural Characteristics and Multiscale Analysis of High-Performance Ionogels

**Fig. 2:**
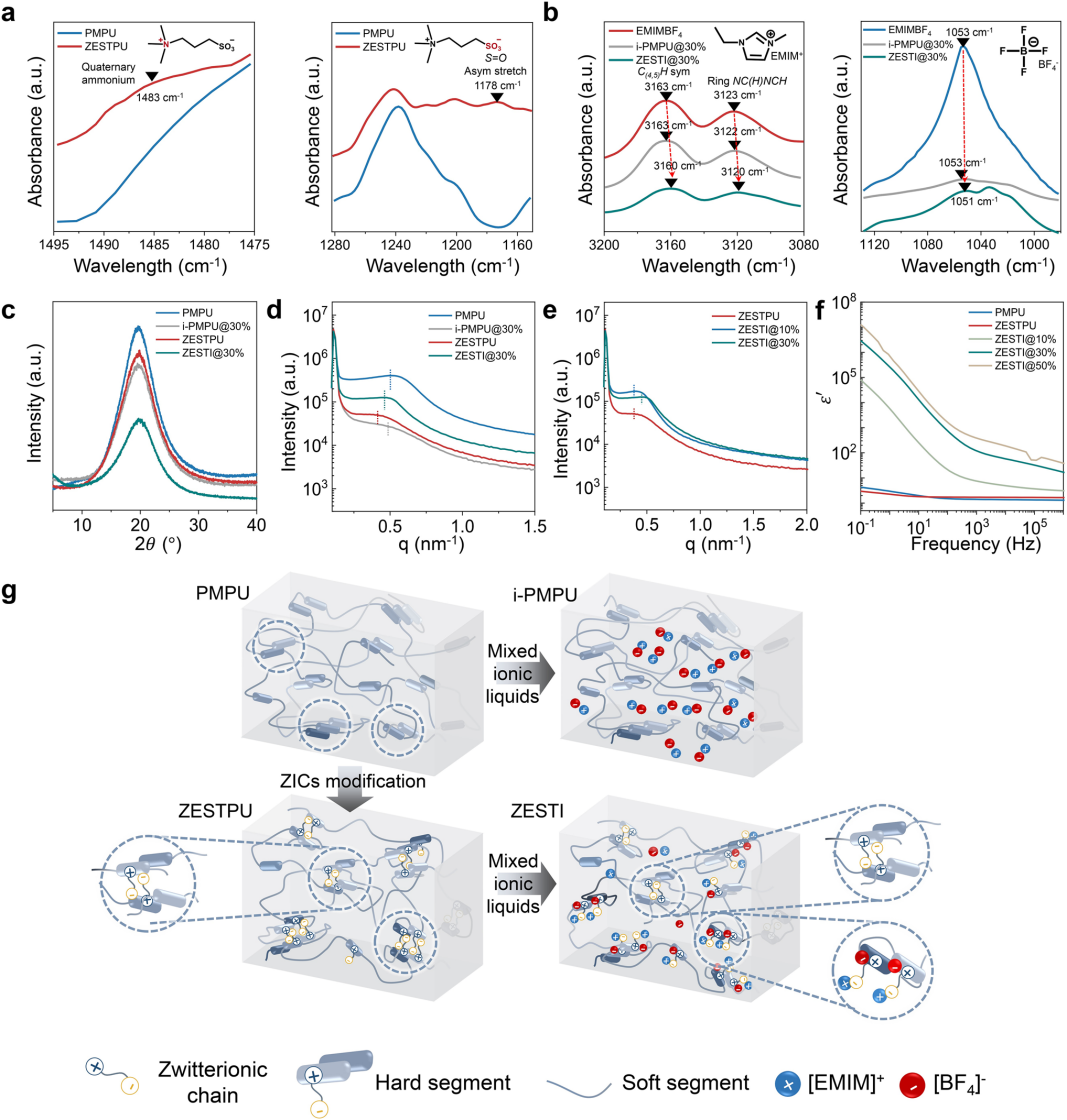
**a** ATR-FTIR spectra of PMPU and ZESTPU confirming the presence of zwitterionic side chains. **b** ATR-FTIR spectra of [EMIM]^+^[BF_4_]^−^, i-PMPU, and ZESTI@30% verifying ion–dipole interactions. **c** XRD patterns and **d, e** SAXS profiles of PMPU, ZESTPU, i-PMPU, and ZESTI. **f** Frequency-dependent dielectric responses of PMPU, ZESTPU, and ZESTIs with different IL contents.** g** Schematic representations of internal structures of PMPU, ZESTPU, i-PMPU, and ZESTI

To verify the successful incorporation of zwitterionic side chains, attenuated total reflectance Fourier transform infrared spectroscopy (ATR-FTIR) was employed to compare the characteristic peaks of PMPU and ZESTPU, as shown in Figs. 2a and S2a. Compared with PMPU, ZESTPU exhibited three new characteristic peaks at 1483, 963, and 1178 cm^−1^, where the former two are assigned to quaternary ammonium groups and the latter to the asymmetric S=O stretching vibration of sulfonate groups [30–32]. Moreover, temperature-dependent FTIR analysis (Fig. S2b) showed that the characteristic peak at 963 cm^−1^ gradually shifted to a higher wavenumber with increasing temperature, suggesting the thermal weakening of dipole–dipole interactions among zwitterionic side chains. These spectral changes confirm the successful incorporation of zwitterionic functionalities into the polyurethane backbone and further suggest the presence of dynamic dipole–dipole interactions among the zwitterionic side chains. In addition, XPS analysis was conducted to further verify the zwitterionic modification, as shown in Fig. S3. Compared with PMPU, ZESTPU showed a distinct S 2*p* signal from sulfonate sulfur in –SO_3_− groups and a shift of the N1s signal to higher binding energy, which is associated with quaternary ammonium formation, confirming the successful incorporation of zwitterionic side chains [33]. Elemental analysis further confirmed the presence of sulfur from the zwitterionic side chains in ZESTPU, supporting their successful incorporation into the polyurethane system (Table S3).

Furthermore, to investigate the ion–dipole interactions between the zwitterionic side chains and the ionic liquid, as well as the corresponding ion dissociation and adsorption behavior, ATR-FTIR spectra of i-PMPU and ZESTI were also performed (Fig. 2b). The characteristic peaks of [EMIM]^+^ corresponding to *C*_(4,5)_*H* symmetric stretching, the NC(H)NCH ring group, and [BF_4_]− were observed at 3163, 3123, and 1053 cm^−1^, respectively. These peaks showed negligible shifts in i-PMPU but exhibited redshifts in ZESTI, indicating that [EMIM]^+^ and [BF_4_]−, respectively, engage in ion–dipole interactions with the zwitterionic side chains [34]. The redshift of the ionic liquid-related FTIR peaks is attributed to the decreased effective force constants of the corresponding vibrational modes. The interaction between ionic liquid and zwitterionic side chains modifies the local electrostatic environment, softens the relevant ionic vibrational modes, and consequently shifts the FTIR peaks toward lower wavenumbers [35, 36]. Consistently, the XPS analysis of ZESTI (Fig. S3e, f) shows that the shift and profile change of the S 2*p* peak indicate an altered local electronic environment of the –SO_3_− groups in the zwitterionic side chains, supporting their interaction with the ionic liquid [33]. Although a high-binding-energy N 1*s* component is also observed at around 401.5 eV, it can be assigned to cationic nitrogen species, including contributions from [EMIM]⁺ and quaternary ammonium groups, so it cannot serve as direct evidence for ion–dipole interactions. The observed peak shifts support specific ion–dipole interactions between the zwitterionic side chains and the ionic liquid. This interpretation is consistent with molecular dynamics studies showing that zwitterionic groups can form stable local associations with mobile ions and regulate ion-coordination environments [37]. In polymer-supported ionic liquid electrolytes, zwitterionic polymer domains have also been shown to influence ion partitioning and transport pathways, including hopping-assisted ion migration [38, 39]. Therefore, the zwitterionic side chains in ZESTI are expected to provide preferential interaction sites for [EMIM]^+^[BF_4_]−, rather than acting as a passive neutral polymer matrix.

To explore the effects of zwitterionic side chains and ionic liquid incorporation on the internal structure of the materials, X-ray diffraction (XRD) and small-angle X-ray scattering (SAXS) were performed to elucidate the structural evolution of the polyurethane matrix, thereby revealing the microstructural basis of high-performance ionogels. As shown in Fig. 2c, all four samples exhibited broad amorphous diffraction halos at similar positions, indicating the absence of crystalline domains or long-range order. Notably, the introduction of the IL did not generate new diffraction peaks, suggesting that it did not disturb the stacking of hard segments. This is attributed to the selective adsorption of the hydrophilic ionic liquid by the hydrophilic zwitterionic chains, which confines the ionic liquid to the hydrophilic domains and prevents its penetration into the hydrophobic hard-segment regions. As a result, the local order of the hard segment is preserved, thereby maintaining their contribution to mechanical strength. The one-dimensional SAXS profiles of the four samples (Fig. 2d) provide further structural insights, with the presence of characteristic scattering peaks confirming the existence of nanoscale microphase-separated structures. The calculated average domain spacing is summarized in Table S4. Compared with PMPU, ZESTPU exhibits a pronounced shift of the scattering peak toward lower angles, indicating an increase in domain size due to the introduction of zwitterionic side chains. This is ascribed to enhanced dipole–dipole interactions between zwitterionic side chains, which promote local aggregation of the hard phase. AFM phase images (Fig. S4) further support the enhanced microphase separation in ZESTPU, which displays distinct bright and dark domains, in contrast to the relatively uniform phase distribution observed in PMPU, where minimal bright features suggest a lower degree of phase separation [14].

Upon introduction of the ionic liquid, the scattering peak shifts toward higher angles, indicating a decrease in average domain spacing. This behavior arises from ion–dipole interactions between the ionic liquid and the zwitterionic side chains, which partially disrupt the aggregated hard phases in ZESTPU and render the phase structure closer to that of the original PMPU. Moreover, Fig. 2e shows that with increasing ionic liquid content, the scattering peak continues to shift to higher angles, reflecting further disassembly of hard phases aggregation and a gradual evolution of the microphase structure toward the unmodified PMPU matrix. Interestingly, when the IL content reaches 30%, the average domain spacing of ZESTI lies between those of PMPU and ZESTPU, suggesting the coexistence of two types of hard-segment structures: (1) enlarged aggregations induced by dipole–dipole interactions among zwitterionic side chains, which contribute to mechanical robustness, and (2) partially disassembled hard phases associated with ionic liquid adsorption, which facilitate ion transport and self-healing behavior. In contrast, the SAXS profile of i-PMPU reveals only a weak shoulder, indicating the loss of distinct microphase separation. This is likely due to the poor compatibility between the hydrophobic polyurethane backbone and the hydrophilic ionic liquid, resulting in macroscopic phase separation beyond the detection range of SAXS [12]. This inference is further supported by the relatively low optical transparency (Fig. 1c) and the deterioration of mechanical properties observed in i-PMPU.

Figure 2f further illustrates the frequency-dependent dielectric response of these materials. To place this phase-structure regulation in the context of previous zwitterionic ionogels, we further compared ZESTI with representative systems in Table S5. In many reported zwitterionic ionogels, zwitterionic motifs primarily serve as ion-coordination sites, ion-transport regulators, or homogeneous network components. In contrast, ZESTI uses zwitterionic side chains to regulate the microphase-separated polyurethane architecture itself. This structural role allows the same molecular motif to reinforce hard-domain cohesion, regulate the distribution of the ionic liquid through preferential ion–dipole interactions, provide reversible ion–dipole interactions for ambient self-healing, and support efficient ionic conduction. Compared to PMPU and ZESTPU, ZESTI with the incorporated ionic liquid exhibits a significantly higher dielectric response across the entire frequency range, with the increase being particularly pronounced in the low-frequency region where ions have sufficient time to migrate. Across the entire graph, the dielectric response increases continuously as the ionic liquid content rises. This indicates that the introduction of ionic liquids significantly increases the amounts of mobile ions capable of participating in polarization within the system, while simultaneously allowing the ionic liquids to form large clusters with amphiphilic ions, thereby creating a larger dielectric. These results suggest that ionic liquids not only help reorganize the microphase-separated structure of polyurethane but also significantly improve the electrical polarizability, providing a crucial foundation for ion transport, self-healing behavior, and device functionality [40]. Figure 2g illustrates the internal structures of PMPU, ZESTPU, i-PMPU, and ZESTI, visually demonstrating how zwitterionic side chains and ionic liquid incorporation regulate the microstructure of the PMPU matrix. This schematic not only clarifies the evolution of the internal morphology but also provides strong support for understanding the structure–property relationships that reflect the performance of the high-performance ionogels developed in our work.

### Mechanical Performance Analysis

**Fig. 3:**
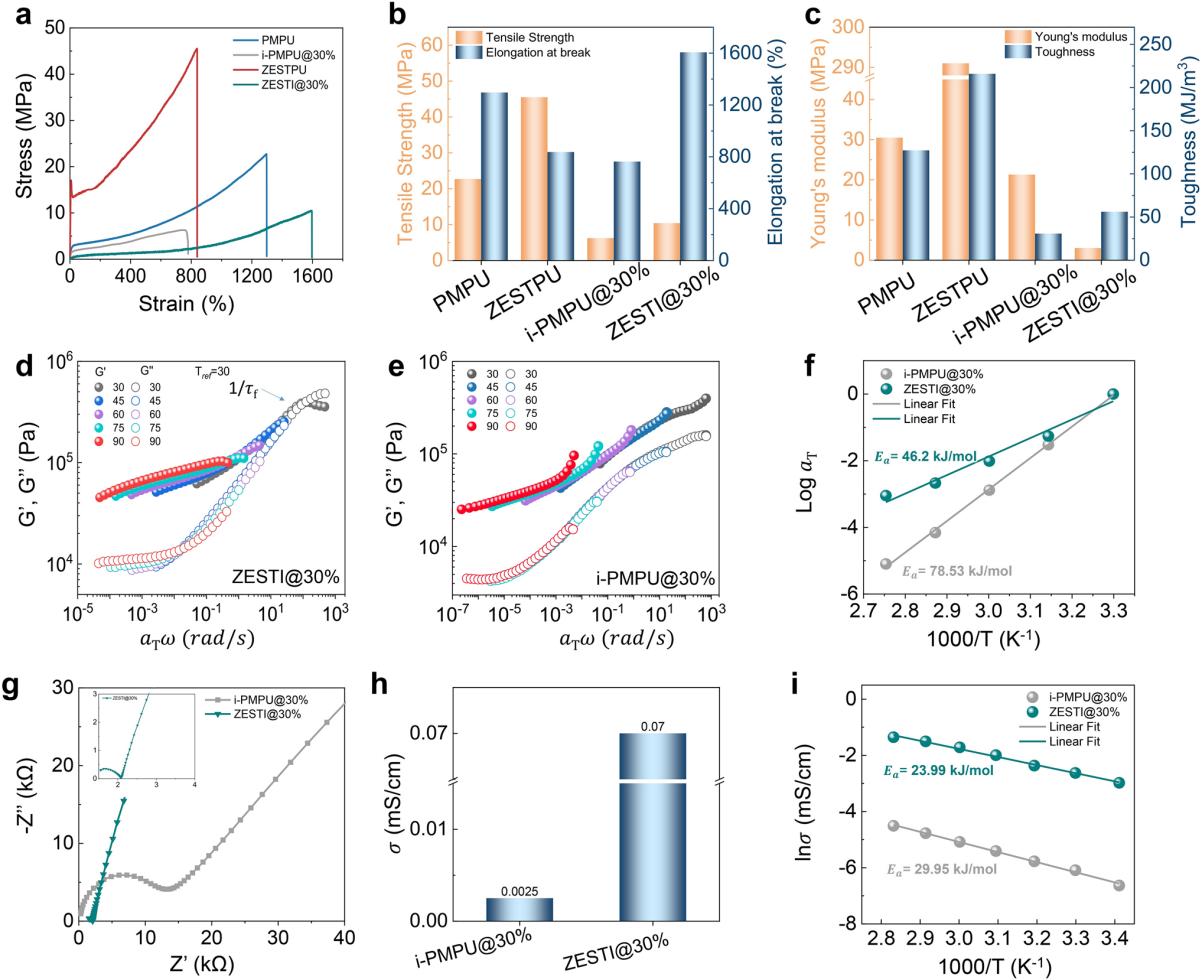
**a** Stress–strain curves of PMPU, ZESTPU, i-PMPU@30%, and ZESTI@30%. **b** Comparison of tensile strength and elongation at break, and **c** comparison of Young’s modulus and toughness for PMPU, ZESTPU, i-PMPU@30%, and ZESTI@30%. Time–temperature superposition rheological curves of the **d** ZESTI@30% and **e** i-PMPU@30% at the reference temperature of 30 °C. **f** Time–temperature horizontal shift factors for i-PMPU@30% and ZESTI@30%. **g** Impedance Nyquist plots, **h** ionic conductivity, and **i** temperature-dependent ionic conductivity analysis of i-PMPU@30% and ZESTI@30%

To investigate the reinforcing effect of zwitterionic side-chain engineering on ionogels (with 30% wt IL), the mechanical performance of four samples was evaluated, as shown in Fig. 3a–c and Table S6. Due to the introduction of zwitterionic side chains, which promote the aggregation of hard-segment domains, ZESTPU exhibited significantly enhanced tensile strength, Young's modulus, and toughness, rising from 22.7 MPa, 30.5 MPa, and 127.2 MJ m^−3^ for PMPU to 45.5 MPa, 291 MPa, and 216.0 MJ m^−3^, respectively. In particular, the substantial increase in modulus clearly indicates that zwitterionic side-chain engineering substantially strengthened the mechanical contribution of the hard phase. Meanwhile, the improved strength of the polyurethane was inevitably accompanied by a reduction in elongation at break, decreasing from 1296% for PMPU to 837% for ZESTPU. This trade-off is mainly attributed to the enhanced aggregation of hard domains, which increases the material rigidity while restricting the mobility and extensibility of the soft segments, thereby reducing the overall stretchability.

Subsequently, the incorporation of ionic liquid led to further variations in the mechanical performance of the resulting ionogel ZESTI compared to ZESTPU. Due to partial dissociation of the aggregated hard domains and a decrease in microphase separation, the tensile strength, Young’s modulus, and toughness of ZESTI@30% declined to 10.40 MPa, 3.0 MPa, and 56.03 MJ m^−3^, respectively. At the same time, the plasticizing effect of the ionic liquid and the weakened strength of the hard phase synergistically increased the elongation at break to 1606%, indicating that the disassembly of the hard network structure released the mobility of polyurethane chains, thereby enhancing the material’s ductility. Although the mechanical strength of ZESTI was lower than that of ZESTPU, it still outperformed most previously reported self-healing ionogels (Fig. 3d). This performance advantage arises from two aspects: the enhanced mechanical integrity originating from the hard-segment aggregation and the dynamic ion–dipole interactions that, despite partial disruption by the ionic liquid, contribute to stress dissipation and provide additional toughness and extensibility. Notably, i-PMPU did not exhibit the typical enhancement in elongation at break commonly observed in conventional ionogels after IL incorporation. Compared with ZESTI, i-PMPU demonstrated significantly lower tensile strength (6.24 MPa), elongation at break (763%), and toughness (30.7 MJ m^−3^), while showing abnormally high modulus (21.3 MPa), indicating excessive rigidity. This result further confirms the earlier hypothesis: Due to the polarity mismatch between the hydrophobic polyurethane backbone and the hydrophilic ionic liquid, severe phase separation occurs in the system, leading to discontinuous polymer–solvent interfaces. As a result, polymer chains are restricted at the phase boundaries and cannot fully disperse into the ionic liquid, thus hindering chain mobility and flexibility recovery, ultimately compromising both ductility and toughness [12].

Given that the mechanical strength of the hard phase is critical for the mechanical performance of the polyurethane matrix, it is necessary to systematically investigate the effect of ionic liquid content. For this purpose, ionogels containing 10%, 30%, and 50% ionic liquid were tested using UTM, as shown in Fig. S5a–c and Table S7. As the ionic liquid content increased, a clear decreasing trend in tensile strength, Young’s modulus, and toughness was observed, owing to the combined effects of plasticization and disruption of the aggregated hard phase. Meanwhile, the elongation at break significantly increased, reflecting enhanced elasticity and deformability of the system. These results further confirm that the integrity and ordering of the aggregated hard phase play a decisive role in achieving high mechanical strength and rigidity in ionogels. Due to the precipitation of ionic liquid observed in the ZESTI@50% sample (Fig. S6), ZESTI@30% was selected as the optimal formulation for subsequent analyses. Unless otherwise specified, ZESTI hereafter refers to ZESTI@30%. In this study, the polyurethane hard/soft segment ratio and zwitterionic grafting condition were kept constant to isolate the role of zwitterionic side chains and their coupled interactions with the ionic liquid. Therefore, the ionic liquid content varied from 10 to 50 wt% as the primary compositional parameter to identify a formulation that balances mechanical integrity, ionic conductivity, and self-healing performance.

For ionogels intended for flexible electronic applications, mechanical toughness and high elongation at break are key determinants of structural durability and device reliability under large deformations and cyclic loading. Therefore, it is essential to explore the structure–property relationship from the perspective of polymer chain dynamics at the microscopic scale to elucidate the origin of macroscopic toughness and extensibility. As shown in Fig. S7a, b, dynamic viscoelastic properties of ZESTI and i-PMPU were measured under 1% strain over a wide frequency and temperature range (30 ~ 90 °C) using a rheometer. The results show that, at lower temperatures, limited chain mobility and enhanced ion–dipole interactions lead to increased storage modulus (*G*′) and loss modulus (*G*″). Furthermore, based on the time–temperature superposition (TTS) principle, master curves of *G*′ and *G*″ over the frequency range of 0.05 ~ 500 rad s^−1^ were constructed at a reference temperature of 30 °C (Fig. 3d, e) [41]. For ZESTI, both *G*′ and *G*″ increase with frequency, exhibiting the typical response of viscoelastic materials. In the plateau region, *G*′ is significantly higher than *G*″, and a crossover point is observed at high frequency, indicating the presence of chain relaxation, slippage, or rearrangement, which facilitates stress release and energy dissipation under large deformation [42]. In contrast, i-PMPU shows a solid-like behavior, with *G*′ remaining higher than *G*″ across the entire frequency range and no crossover point observed. This suggests that chain mobility is restricted and that the system lacks effective energy-dissipation mechanisms, resulting in greater rigidity and reduced flexibility.

This conclusion is further supported by stress relaxation experiments. As shown in Fig. S7c, under the same constant strain (1%), ZESTI exhibits rapid stress decay of approximately 80% within 50 s, stabilizing below 1/*e*, indicating a significantly faster relaxation capacity compared to i-PMPU. The decay behavior of the stress–time curve reflects time-dependent slippage and rearrangement of polymer chains at room temperature, further demonstrating excellent network reconfigurability [43]. To further quantify the polymer chain dynamics in ionogels, horizontal shift factors (*a*_*T*_) obtained during the construction of the master curves were extracted and plotted as a function of temperature (Fig. 3f), which follows an Arrhenius relationship (Eq. 1) [41, 44]:

1 $${\mathrm{a}}_{T} = A\exp \left( { - \frac{{E_{a} }}{RT}} \right)$$

where *a*_*T*_ is the horizontal shift factor in the time–temperature superposition rheological curves, *A* is the pre-exponential factor, *E*_*a*_ is the activation energy, *R* is the ideal gas constant, and *T* is the Kelvin temperature. Fitting the curves yielded apparent activation energies of 46.2 kJ mol^−1^ for ZESTI and 78.5 kJ mol^−1^ for i-PMPU. The higher activation energy indicates a greater restriction on polymer chain mobility in i-PMPU, primarily due to stronger phase separation between the polymer matrix and the ionic liquid, which makes it more difficult for the chains to overcome cohesive energy barriers for effective relaxation under thermal excitation.

Finally, to evaluate the cyclic mechanical stability of ZESTI under repeated deformation, cyclic tensile loading–unloading tests were performed over a strain range of 0–300%. As shown in Fig. S8a, the first cycle exhibited pronounced hysteresis, which is mainly associated with ionic interactions, dynamic physical cross-linking, and viscoelastic relaxation of the polymer network. After the initial cycle, the 2nd-10th cycles showed highly overlapped curves, and the maximum stress at 300% strain remained stable at approximately 1.3–1.4 MPa. These results demonstrate that ZESTI possesses good elastic recovery and cyclic mechanical stability, supporting its reliable sensing performance during long-term use.

To further elucidate the strain-dependent energy-dissipation behavior, cyclic loading–unloading tests were conducted at different maximum strains of 100%, 200%, 400%, and 600% (Fig. S8b). The enlarged hysteresis loops with increasing strain indicate that ZESTI dissipates more mechanical energy under larger deformation. Quantitative analysis showed that *U*_*diss*_ increased from 0.254 MJ m^−3^ at 100% strain to 3.858 MJ m^−3^ at 600% strain, while the hysteresis ratio only slightly increased to 57.7% (Fig. S8c and Table S7). This relatively stable hysteresis ratio suggests that the increased *U*_diss_ is not dominated by irreversible network damage, but mainly arises from the repeated dissociation and reformation of reversible interactions. This behavior is consistent with the zwitterionic side-chain engineering strategy, where dipole–dipole interactions reinforce the polyurethane physical cross-linking domains, while dynamic ion–dipole interactions provide reversible dissipation sites during stretching. Consequently, ZESTI can effectively dissipate mechanical energy without sacrificing network integrity, contributing to its high toughness of 56.03 MJ m^−3^.

### Electrical Performance Analysis

To systematically evaluate the electrical performance of the ionogels and further investigate the influence of zwitterionic side-chain enabled ion channels on ion transport behavior, EIS measurements were performed, as shown in Figs. 3g–h and S9a–b. The results revealed a significant improvement in ionic conductivity with increasing ionic liquid content. Notably, ZESTI@30% exhibited a markedly higher conductivity of 0.07 mS cm^−1^ compared to the control sample i-PMPU@30% (0.0025 mS cm^−1^), which lacks zwitterionic channels. This pronounced improvement highlights the crucial role of zwitterionic side chains in constructing efficient ion transport pathways within the polymer network [45]. Recent studies on zwitterionic and supramolecular ionic gels have shown that polar ionic motifs can regulate local ion coordination, promote ionic liquid dissociation, and construct preferential ion-transport pathways [46, 47]. This interpretation is consistent with our ZESTI system, in which ion–dipole interactions between zwitterionic side chains and [EMIM]^+^[BF_4_]^−^ facilitate ion hopping and lower the migration barrier [25, 28, 29, 48, 49]. The reduced activation energy of ZESTI compared with i-PMPU further supports that zwitterionic side chains promote ion transport rather than merely increasing the ionic liquid content. To gain a deeper mechanistic understanding of the enhanced ion transport, particularly in terms of chain mobility and energy barrier modulation, the temperature-dependent ionic conductivity of i-PMPU and ZESTI was systematically examined. The relationship between the logarithm of conductivity (ln σ) and the inverse of temperature (1/T) in the range of 293.15 ~ 353.15 K was plotted, as shown in Fig. 3i. The results reveal that the ionic conductivity of both materials increases with temperature and follows the Arrhenius behavior (Eq. 2) [50]:

2 $$\sigma = \sigma_{0} \exp \left( { - \frac{{E_{a} }}{RT}} \right)$$

where *E*_*a*_ represents the activation energy, σ is the ionic conductivity, σ_0_ is the pre-exponential factor, *T* is the absolute temperature, and *R* is the universal gas constant. Linear fitting curves reveal that the ZESTI exhibits a notably lower *E*_*a*_ (23.99 kJ mol^−1^) compared to i-PMPU (29.95 kJ mol^−1^), indicating a reduced energy barrier for ion migration. This reduction arises from the dual function of the zwitterionic side chains, which both facilitate ionic liquid dissociation and in guide ion migration pathways under an applied electric field, thereby substantiating their critical role in enhancing ionic conductivity [48, 49].

In this system, the formation of ion channels is intimately linked to the dissociation behavior of ionic liquids within the zwitterionic aggregation domains, with the channel density being directly governed by the ionic liquid content. As shown in Fig. S10, the ZESTI@10% reveals a notably higher activation energy (35.8 kJ mol^−1^) compared to ZESTI@30%. This suggests that insufficient ionic liquid limits the formation of efficient ion-transport pathways, thereby increasing the migration barrier and reducing ionic conductivity by restricting ion mobility within the polymer network.

### Autonomous Self-Healing Behavior and Property Analysis

**Fig. 4:**
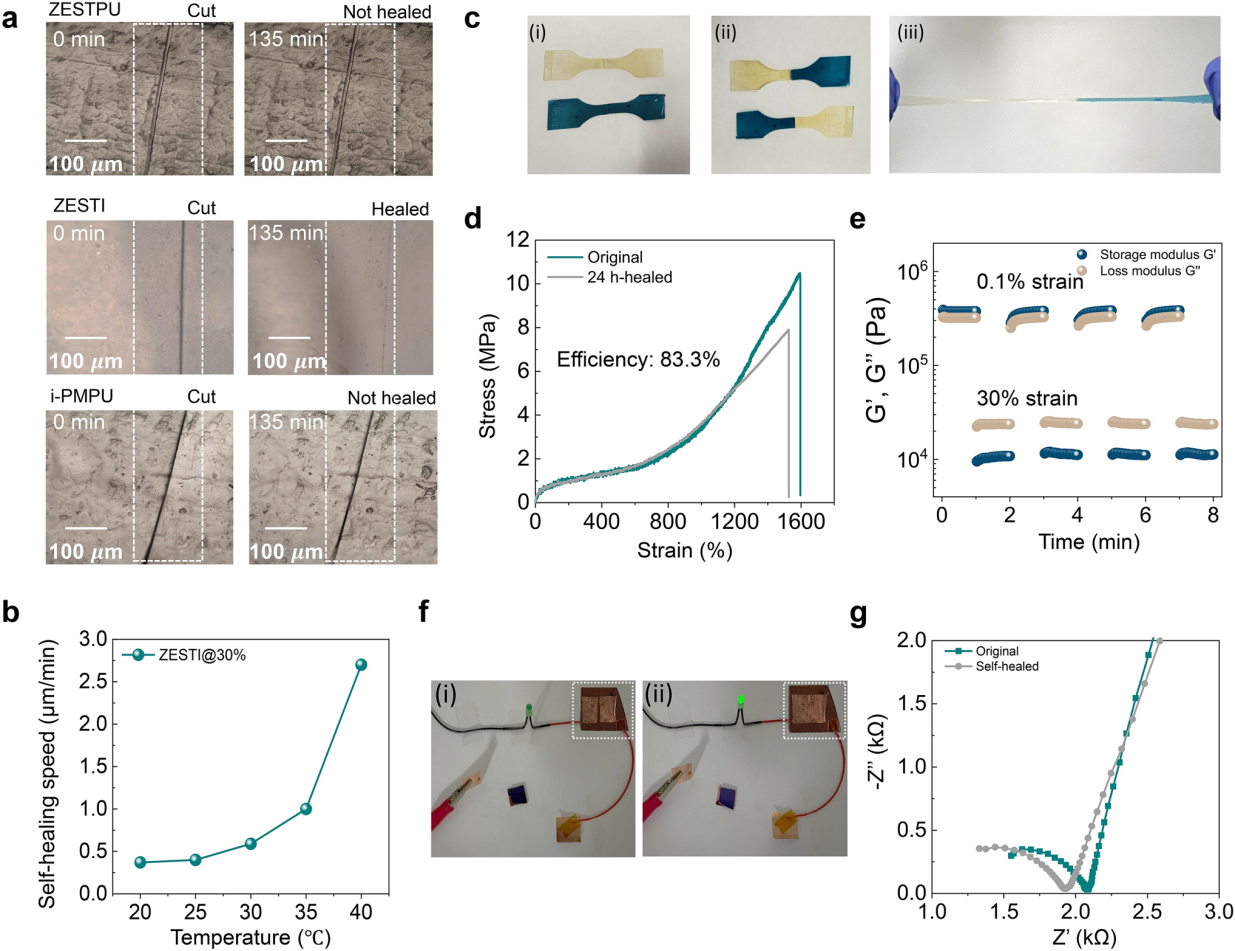
**a** Optical microscope images showing autonomous self-healing of surface scars in ZESTPU, ZESTI, and i-PMPU at room temperature (scale bar: 100 µm). **b** Self-healing speed of ZESTI at different temperatures. **c** Two individually cut and rejoined ZESTI films successfully healed and stretched without failure. **d** Stress–strain curves of original and self-healed ZESTI films. **e** Oscillatory time sweep of ZESTI at 25 °C. **f** Photographs of an LED circuit before and after self-healing of the ZESTI-based conductive path. **g** Impedance Nyquist plots of ZESTI before and after self-healing. The environmental stability of ZESTI was further examined by measuring its ionic conductivity under varying humidity and during prolonged exposure to RH 90%. As shown in Fig. S11, the ionic conductivity remained stable across the measured humidity range and was largely maintained under RH 90%, confirming reliable ionic performance under environmentally relevant humidity.

Compared to ZESTPU and i-PMPU, ZESTI exhibited excellent self-healing capabilities at room temperature, primarily due to the abundant ion–dipole interactions present in the system. As shown in Fig. 4a, scratch healing experiments were performed under an optical microscope with an initial scratch depth of ~ 50 μm. After 135 min, the scratch on ZESTI had completely disappeared, confirming its intrinsic self-healing capability. In contrast, ZESTPU and i-PMPU, lacking dynamic ion–dipole interactions, showed no noticeable healing over the same duration, with their scratches remaining clearly observable. To quantify the healing kinetics, the time required for complete scratch disappearance at various temperatures was recorded, and the corresponding self-healing speed (Fig. 4b) was calculated based on Eq. 3 [51, 52]:

3 $${\text{Self{-}healing}}\;{\mathrm{speed}} = \frac{{{\mathrm{Thickness}}\;(\mu {\mathrm{m)}}}}{{{\text{Self{-}healing}}\;{\mathrm{time}}\;({\mathrm{min)}}}}$$

The results revealed a clear acceleration in self-healing speed with increasing temperature, attributed to enhanced polymer chain mobility and the dynamic nature of ion–dipole interactions that facilitate faster molecular rearrangement and interface recovery at elevated temperatures. Consistent with this temperature dependence, autonomous self-healing in ZESTI proceeds efficiently under ambient and moderately elevated temperatures, whereas it is strongly limited at sub-zero temperatures, where the reduced polymer chain mobility and slowed reconfiguration of ion–dipole interactions restrict interfacial reconstruction. The demonstrated autonomous self-healing of ZESTI is therefore mainly applicable under ambient conditions, which correspond to the typical operating environment of skin-like and wearable iontronic devices. Self-healing of mechanical properties is particularly crucial for ionogels in practical applications, where long-term durability and reliability are essential. As illustrated in Fig. 4c, dog-bone-shaped ZESTI samples dyed in different colors were cut and manually reassembled in a staggered arrangement. After healing at room temperature for 24 h, the rejoined samples exhibited good stretchability without visible failure, preliminarily demonstrating self-healing ability. Subsequent quantitative analysis using a UTM (Fig. 4d) further validated this observation, yielding a self-healing efficiency of 83.3%.

To further explore the dynamic reconfiguration capability of ZESTI, amplitude and time sweep rheological experiments were performed (Figs. S12 and 4e). In the amplitude sweep test, the G′ exceeded the G″ in the low-strain region and intersected at approximately 5.5% strain, beyond which G′ sharply declined below G″, signifying network disruption. In the subsequent time sweep experiment, alternating cyclic strains of 0.1% and 30% were applied, during which G′ dropped below G″ at 30% strain, confirming disruption of the network structure. However, upon returning to 0.1% strain, both moduli rapidly recovered to near their initial values, with G′ again surpassing G″, clearly demonstrating the excellent dynamic reconstruction and self-healing capability of the ZESTI network [53, 54].

Furthermore, considering the potential applications of ionogels in flexible electronic sensors, the recovery of electrical performance after mechanical damage is crucial. As shown in Fig. 4f, ZESTI was integrated into a metal–ionogel–metal (MIM) structure connected to an LED and an AC power source. When the sample was cut, the LED turned off. Upon reconnection, the circuit was restored and the LED re-illuminated, indicating the re-establishment of the conductive pathway. To validate this observation, LCR measurements were performed before and after cutting (Fig. S13), confirming the restoration of electrical functionality. Further evidence was provided by EIS (Fig. 4g), which revealed nearly overlapping impedance spectra before and after healing, demonstrating that ZESTI reliably recovers its electrical properties following mechanical damage. The above structural and functional analyses establish a direct nano-/microstructure–property relationship that governs the device-level performance of ZESTI. The zwitterionic side chains organize the ionogel into a microphase-separated architecture in which mechanically reinforcing hard domains, dynamic ion–dipole interaction sites, and ion-conducting regions coexist within a single matrix. This structural organization is essential for iontronic operation. The reinforced hard domains provide mechanical tolerance against puncture, compression, and repeated deformation, while the dynamic ion–dipole interactions allow damaged interfaces and interrupted ionic pathways to be reconstructed autonomously. At the same time, preferential interaction between the zwitterionic domains and ionic liquid maintains continuous ion-transport pathways, enabling stable impedance readout and strain-dependent electrical responses. Therefore, the device demonstrations below are not merely applications of a high-performance ionogel, but direct consequences of the nanoscale phase organization and dynamic interaction network engineered in ZESTI.

To connect the material properties of ZESTI with device-level functions, we selected two complementary iontronic demonstrations that require stable ionic conduction under mechanical damage and deformation. The self-reporting packaging interface evaluates ZESTI under localized mechanical stress, where sharp contact, compression, and damage can interrupt ionic pathways and destabilize electrical readout. The strain sensor evaluates the same material platform under distributed tensile deformation, where stretchability, repeatable resistance response, and post-damage recovery are required. Thus, these demonstrations are not separate applications, but two operating scenarios that validate the same material advantages of ZESTI, including mechanical robustness, dynamic ionic pathway reconstruction, and autonomous recovery of electrical function.

### Self-Healable Ionogel-Based Damage-Tolerant, Self-Reporting Interface

**Fig. 5:**
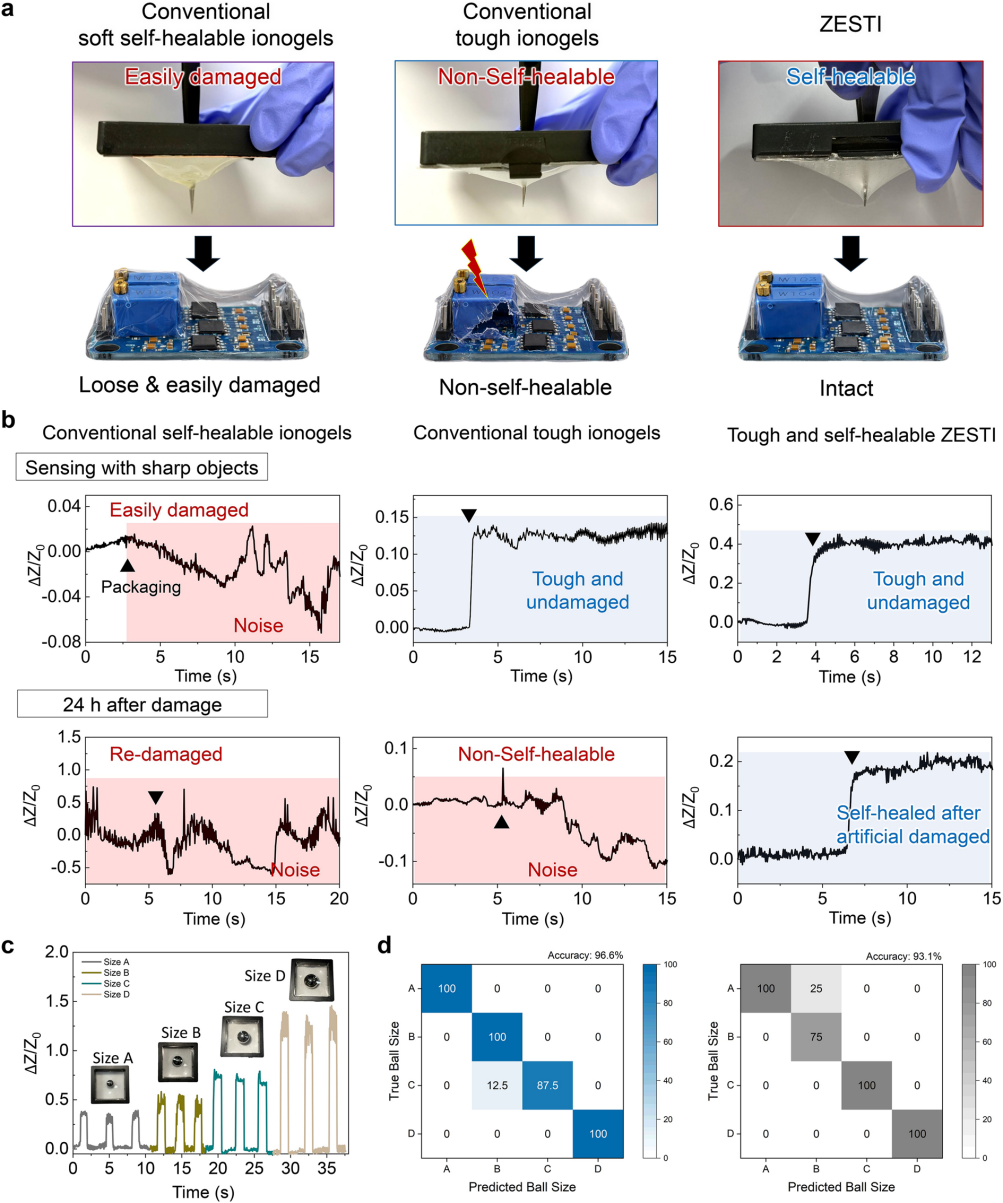
**a** Conceptual illustration of device packaging using ionogel protective interface, comparing conventional soft self-healable ionogels, conventional tough ionogels, and ZESTI. ZESTI enables conformal protection of devices with protruding structures while maintaining structural integrity. **b** Real-time impedance response under sharp object-induced mechanical damage. ZESTI maintains stable sensing signals and recovers after damage, whereas conventional ionogels show instability or irreversible failure. **c** Demonstration of compression-dependent impedance responses for objects with different diameters (14 mm, 19 mm, 22 mm, and 25 mm), illustrating the capability of the ZESTI interface to convert mechanical packaging states into distinguishable electrical signals. **d** Confusion matrices for size classification using the impedance signals obtained from the ionogel interface, showing high classification accuracy and reliable identification of different object sizes

In auto-/semi-automated packaging processes, a critical requirement is not only to provide mechanical protection but also to enable real-time assessment of whether devices are properly compressed, damaged, or subjected to abnormal mechanical conditions. However, conventional packaging materials typically offer only passive mechanical support and lack the capability for state feedback and damage recovery. As shown in Fig. 5a, conventional soft self-healable ionogels lack the mechanical robustness required to withstand sharp structures, which often causes structural failure, unstable fixation, and device damage. In contrast, although conventional tough ionogels can initially resist such mechanical damage, they lack self-healing capability and therefore cannot recover once torn or punctured. This irreversible damage fundamentally limits their durability and prevents sustainable long-term use. To address these limitations, we propose a damage-tolerant, self-reporting interface based on the ZESTI, which enables reliable mechanical protection while simultaneously providing real-time feedback on the packaging state and damage conditions. We emphasize that this interface is presented as a proof-of-concept validation of the ZESTI material concept rather than a fully optimized packaging product, and its operation directly reflects the same material advantages established above, including mechanical damage tolerance from the reinforced hard domains, dynamic reconstruction of interrupted ionic pathways, and recovery of the impedance readout after damage. Owing to its mechanical stability and interfacial adaptability, ZESTI maintains structural integrity during the packaging of objects with sharp features and achieves long-term conformal contact with protruding structures.

We further evaluated its sensing stability under dynamic packaging conditions (Fig. 5b and Videos S1–S3). Under sharp object interaction, conventional self-healable ionogels are prone to catastrophic mechanical rupture, which induces erratic signal fluctuations and a total loss of sensing fidelity. Even after self-healing, their insufficient mechanical properties fail to support stable sensing behavior. In comparison, conventional tough ionogels exhibit stable responses under low-stress conditions but undergo irreversible damage under higher stress. Due to the absence of self-healing capability, their sensing function cannot be recovered. In contrast, ZESTI leverages its dynamically damage-tolerant and self-reporting interface to maintain stable sensing responses across a wide range of object sizes and sharp contact conditions without structural failure. Moreover, after artificial damage, the ZESTI restores continuous ionic transport pathways through self-healing, thereby recovering stable impedance signals. This behavior demonstrates its excellent sensing stability and long-term durability under mechanically challenging conditions.

Building upon this, we further investigated the size recognition capability of the protective interface. As shown in Fig. 5c, objects of different sizes induce distinct interfacial deformation during packaging, leading to distinguishable impedance responses for accurate size identification. The perceptive packaging box accommodates stainless steel spheres of various sizes without tearing while preserving clear signal separation, confirming its robustness and reliability. To further assess its practical applicability, we evaluated the post-healing performance stability. As shown in Fig. S14, the impedance responses exhibit excellent reproducibility before and after self-healing, indicating that the system can effectively recover its sensing functionality and maintain stable long-term operation. To quantitatively assess the size recognition capability, we constructed confusion matrices based on the impedance signals. As shown in Figs. 5d and S15, the classification accuracies reach 96.6% and 93.1% before and after self-healing, respectively. These results demonstrate the high sensitivity of ZESTI to small deformations and provide proof-of-concept validation that its mechanical damage tolerance and self-healing-enabled recovery of ionic pathways translate into a functional, damage-tolerant sensing interface.

### Self-Healable Ionogel-Based Strain Sensing

After demonstrating localized damage tolerance and self-reporting capability in the packaging interface, we further evaluated ZESTI under distributed tensile deformation using a stretchable strain sensor. This second demonstration was designed to verify whether the same self-healable ionic network can maintain stable signal transduction during repeated stretching and human motion monitoring under daily ambient conditions. As shown in Fig. S16a, the relative resistance change (ΔR/R₀) of the sensor increased monotonically with applied strain, exhibiting two distinct linear regions in the strain-resistance response curve. The calculated gauge factors (GF) were 1.44 and 2.07 in the 0–250% and 250%–500% strain ranges, respectively, indicating reliable strain sensitivity over a wide deformation range. To further assess the real-time electrical response under varying strain conditions, relative resistance changes from 0 to 100% strain were monitored. As shown in Fig. S16b, under strain levels of 1%, 3%, 5%, 10%, 20%, 50%, and 100%, the sensor exhibited clear and distinguishable signal responses, with increasing signal amplitude correlating with strain magnitude, demonstrating both high sensitivity and excellent strain resolution. Additionally, the sensor’s long-term durability was evaluated by subjecting it to 1000 cycles of stretching at 50% strain (Fig. S16c). The results showed negligible signal degradation, confirming outstanding mechanical stability and operational reliability under repeated deformation. Moreover, the ZESTI-based strain sensor exhibits fast response and recovery under 50% strain at 1000 Hz, with response and reset times of 49 and 48 ms, respectively, further confirming its suitability for real-time strain monitoring (Fig. S16d). Furthermore, the ΔR/R₀ strain loading–unloading curves exhibited nearly overlapping profiles during stretching and releasing, and the calculated hysteresis ratio was only 2.55%, confirming the low electromechanical hysteresis and highly reversible signal transduction of the ZESTI-based strain sensor under tensile deformation (Fig. S16e).

To explore the sensor’s applicability in real-time human motion monitoring, a series of biomechanical signal detection tests were performed. As illustrated in Fig. S17a, the sensor was affixed to a finger joint, where relative resistance changes under different bending angles (0°, 30°, and 90°) could be clearly distinguished. Similarly, when attached to the cheek area (Fig. S17b), the sensor effectively responded to facial expressions such as smiling and laughing, outputting distinguishable resistance signals. The device also demonstrated reliable detection of joint movements at the elbow, accurately differentiating between bending angles of 0°, 30°, and 90° (Fig. S17c), further verifying its responsiveness to large-range joint deformation. Furthermore, the sensor’s long-term performance under continuous dynamic monitoring was evaluated via a 1000-s elbow bending test (Fig. S17d). The sensor exhibited stable signal output with excellent repeatability and no significant drift or degradation, thereby confirming the ZESTI-based strain sensor’s robustness and practical potential in complex dynamic environments.

## Conclusion

In summary, we have developed a zwitterionic side-chain engineered tough ionogel that effectively overcomes the long-standing trade-offs between mechanical robustness, ionic conductivity, and autonomous self-healing in soft ionic materials. By incorporating hydrophilic zwitterionic side chains into hydrophobic polyurethane hard segments, ZESTI maintains well-defined microphase separation while reinforcing the cohesive integrity of the hard domains. This molecular architecture enables dynamic ion–dipole interactions that significantly enhance mechanical elasticity and energy dissipation, yielding a high tensile strength of 10.4 MPa, remarkable elongation of 1606%, and exceptional toughness of 56.03 MJ m^−3^. Meanwhile, the zwitterionic moieties facilitate efficient ion hopping, resulting in markedly improved ionic conductivity. Critically, the intrinsic segmental mobility of the zwitterionic side chains enables spontaneous self-healing under ambient conditions, achieving over 83% recovery efficiency without external stimuli. To demonstrate its practical properties, ZESTI was implemented as a multifunctional encapsulation platform to deliver stable electrical readout under severe deformation and sharp surface abrasion. Even after surface-induced damage, ZESTI autonomously restores mechanical and electrical functionality via spontaneous self-healing, enabling repeatable operation under mechanically harsh conditions. These proof-of-concept demonstrations establish ZESTI as a promising multifunctional, sustainable ionogel platform for next-generation wearable electronics, human–machine interfaces, and soft bioelectronic systems demanding high durability, reversibility, and stable ionic performance under real-world conditions.

## Supplementary Information

Below is the link to the electronic supplementary material.

Supplementary file1 (DOCX 9484 kb)

Supplementary file2 (MP4 5615 kb)

Supplementary file3 (MP4 5792 kb)

Supplementary file4 (MP4 6000 kb)
