# Supplementary material for: Zwitterionic Ionogels Resolving the Trade-Off Between Mechanical Strength and Autonomous Self-Healing for Iontronics
Source: Nanomicro Lett. 2026 Jul 30;19:11. doi: 10.1007/s40820-026-02318-1 (PMC13424064; doi:10.1007/s40820-026-02318-1)
Supplement: Supplementary file 1 — Supplementary file1 (DOCX 9484 kb) [file 40820_2026_2318_MOESM1_ESM.docx]

Supporting Information for

**Zwitterionic Ionogels Resolving the Trade-Off between Mechanical Strength and Autonomous Self-Healing for Iontronics**

Zhengyang Kong^1^†, Ji Hong Kim^1^†, Jonghwi Kim^1^, Woojin Lee^1^, Hayoung Oh^1^, Wu Bin Ying^2^, Joo Sung Kim^3^, Seonghwan Yun^1^, So Young Kim^1^, Do Hwan Kim^1,4,*^

Department of Chemical Engineering, Hanyang University, Seoul 04763, Republic of Korea

^2^School of Electrical Engineering (EE), Korea Advanced Institute of Science and Technology (KAIST), Daejeon 34141, Republic of Korea

^3^Thin-Film Device Laboratory & Center for Emergent Matter Science (CEMS), RIKEN, 2-1 Hirosawa, Wako, Saitama 351-0198, Japan

4Institute of Nano Science and Technology, Hanyang University, Seoul 04763, Republic of Korea.

†Zhengyang Kong and Ji Hong Kim contributed equally to this work.

*Corresponding author. E-mail: [dhkim76@hanyang.ac.kr](mailto:dhkim76@hanyang.ac.kr) (Do Hwan Kim)

**Supplementary Figures and Tables**


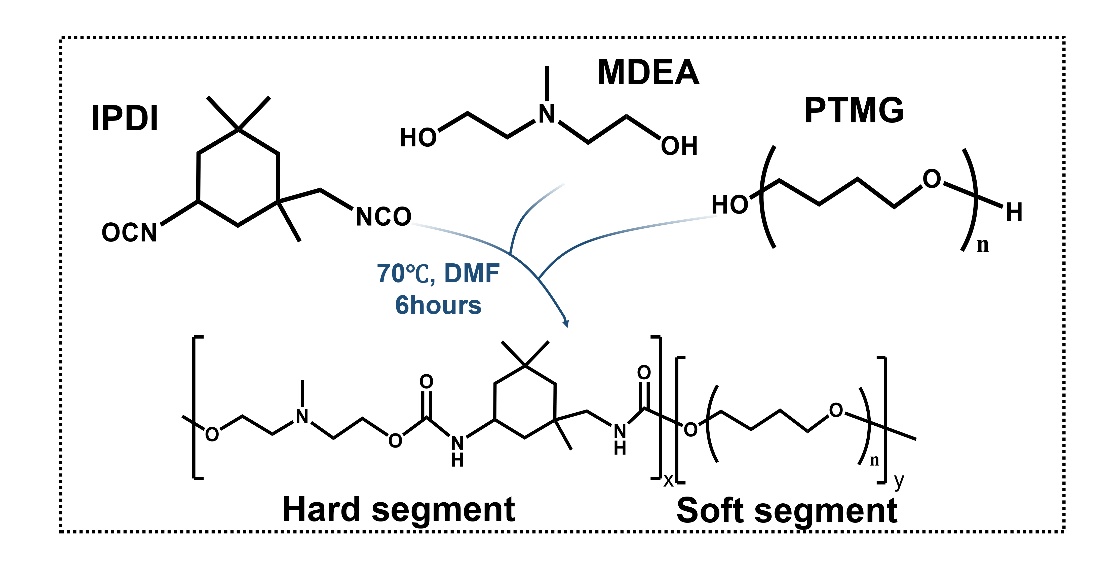


**Scheme S1** Schematic synthetic route of PMPU


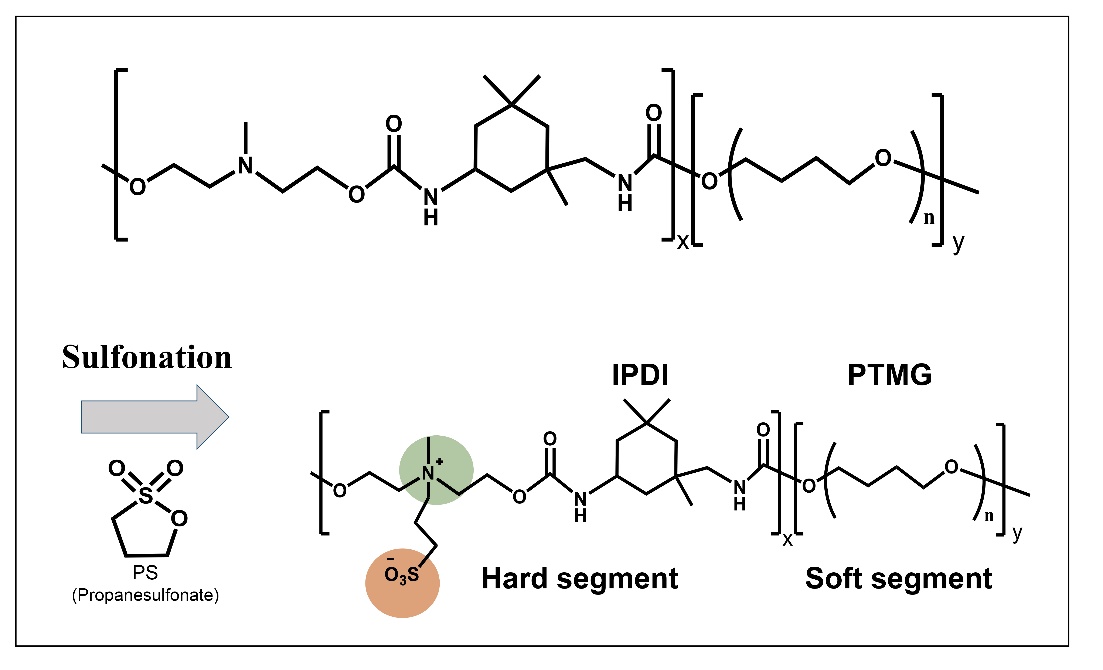


**Scheme S2** Schematic synthetic route of ZESTPU

**
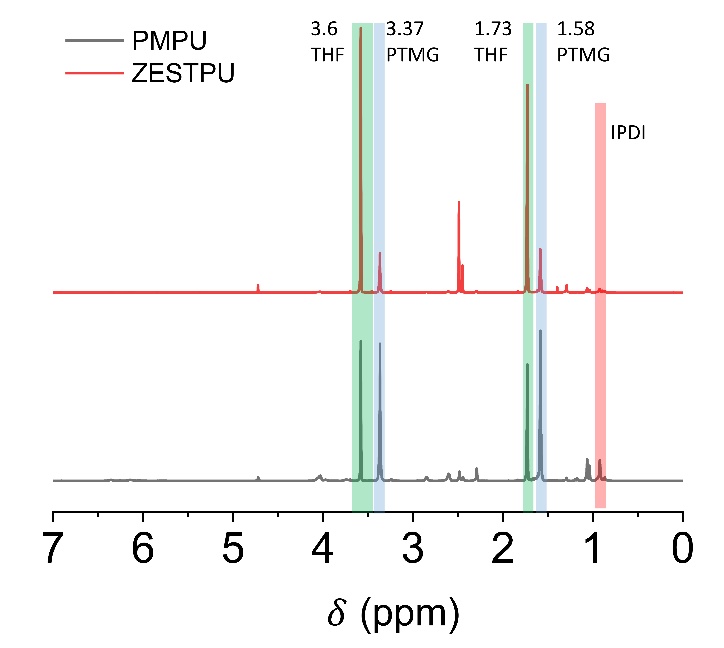
**

**Fig. S1** ^1^H NMR characterization of PMPU and ZESTPU.


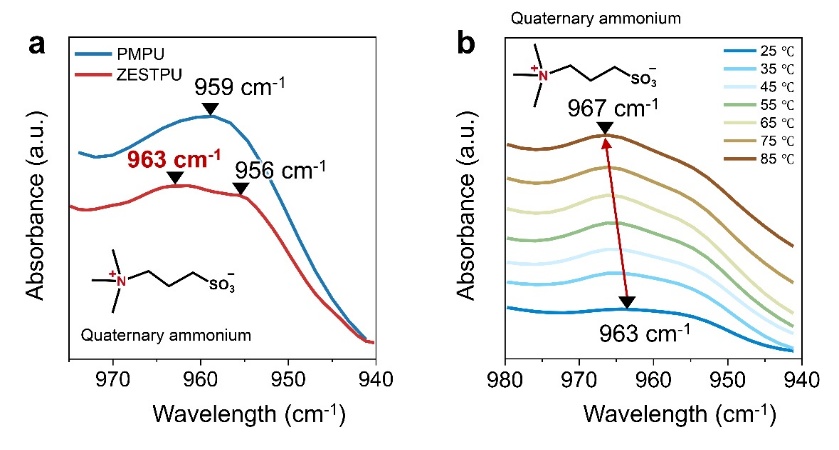


**Fig. S2** (a) ATR-FT-IR spectra of PMPU and ZESTPU confirming the presence of zwitterionic side chains. (b) Variable-temperature FT-IR spectra of ZESTPU verifying dipole-dipole interactions.


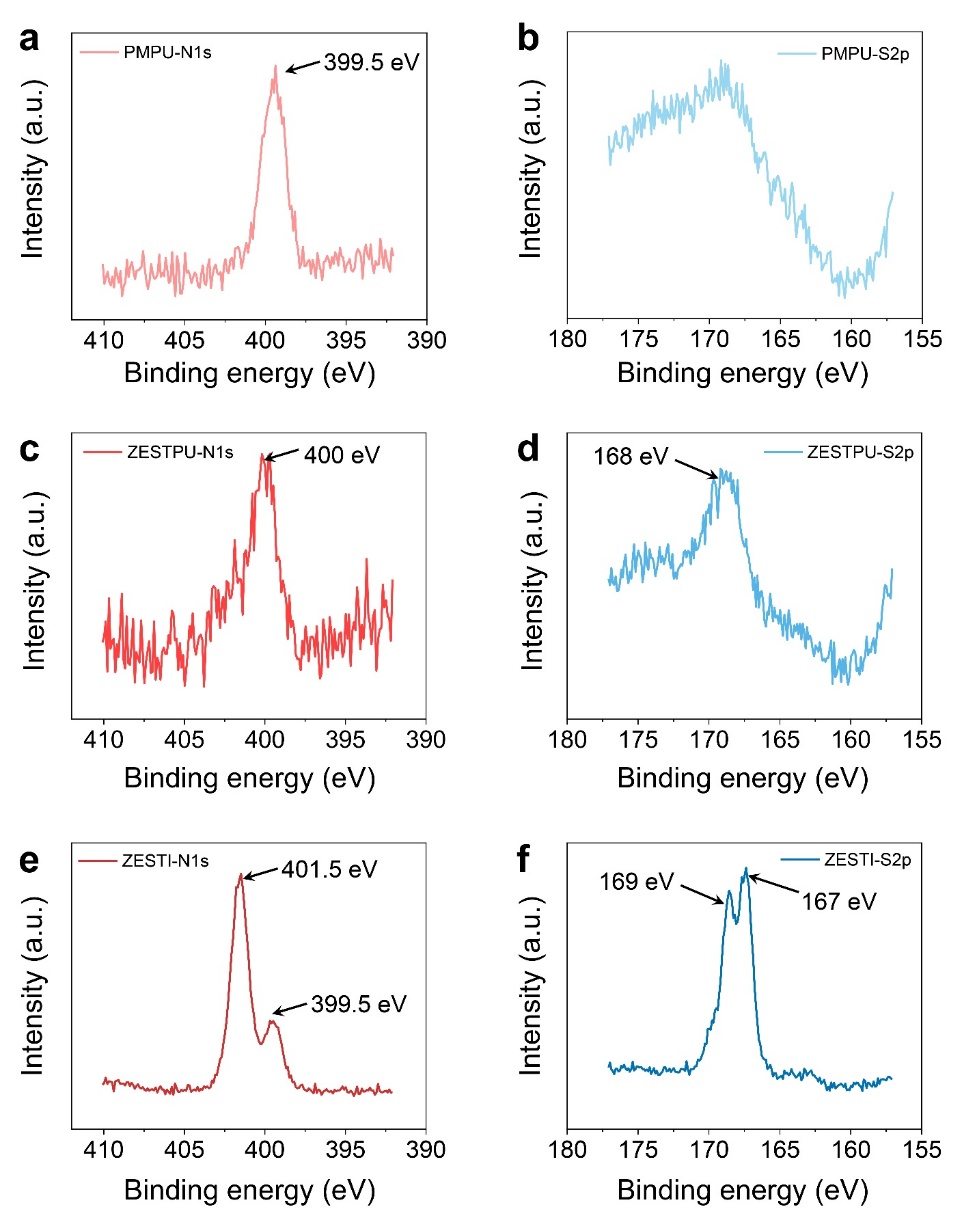


**Fig. S3** High-resolution XPS spectra of (a, b) PMPU, (c, d) ZESTPU, and (e, f) ZESTI. Panels (a), (c), and (e) show the N 1s spectra, while panels (b), (d), and (f) show the S 2p spectra.


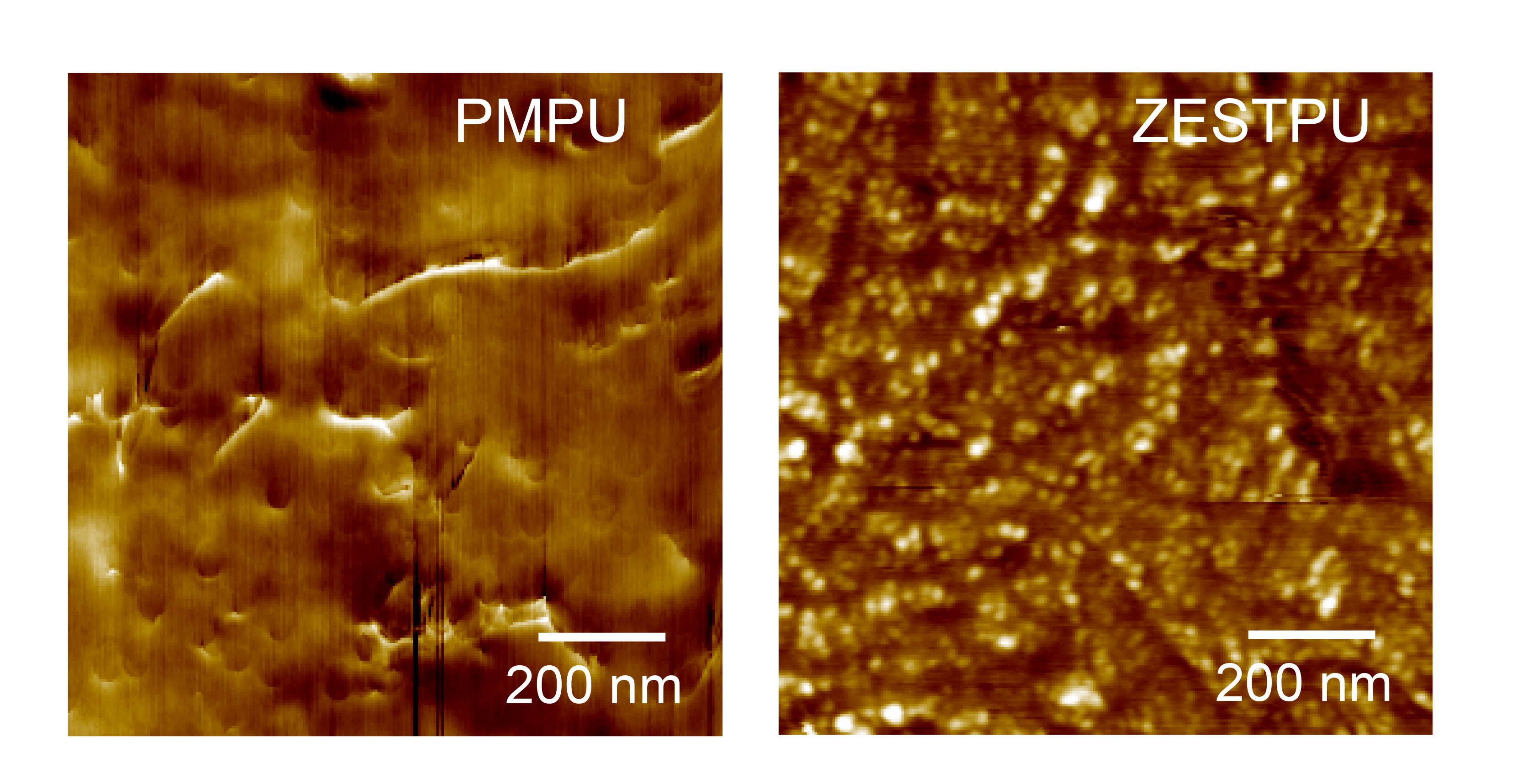


**Fig. S4** AFM phase image of PMPU and ZESTPU.


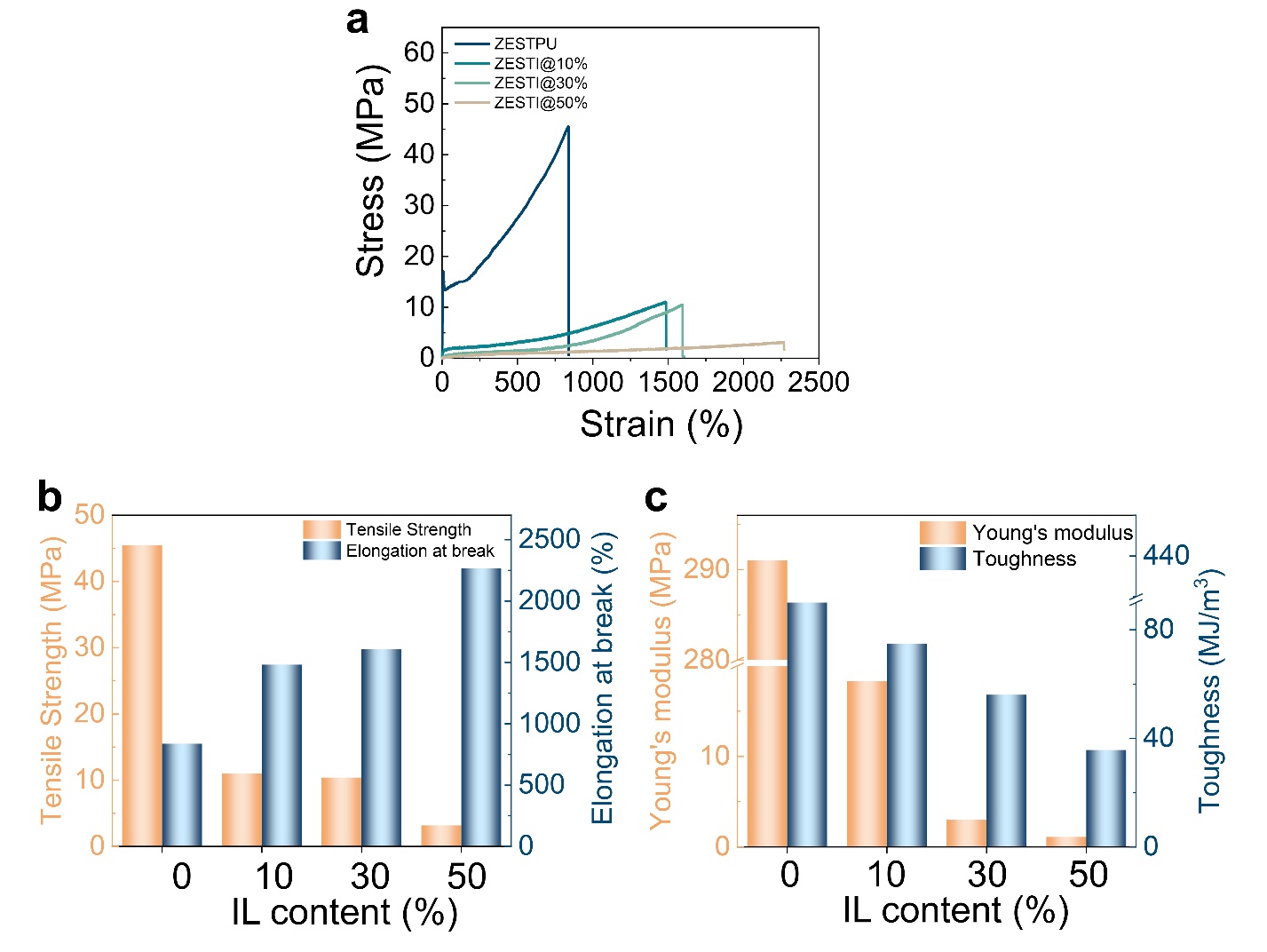


**Fig. S5** (a) Stress-strain curves of ZESTPU with different IL contents. (b) Comparison of tensile strength and elongation at break, and (c) comparison of Young’s modulus and toughness for ZESTPU with different IL contents.


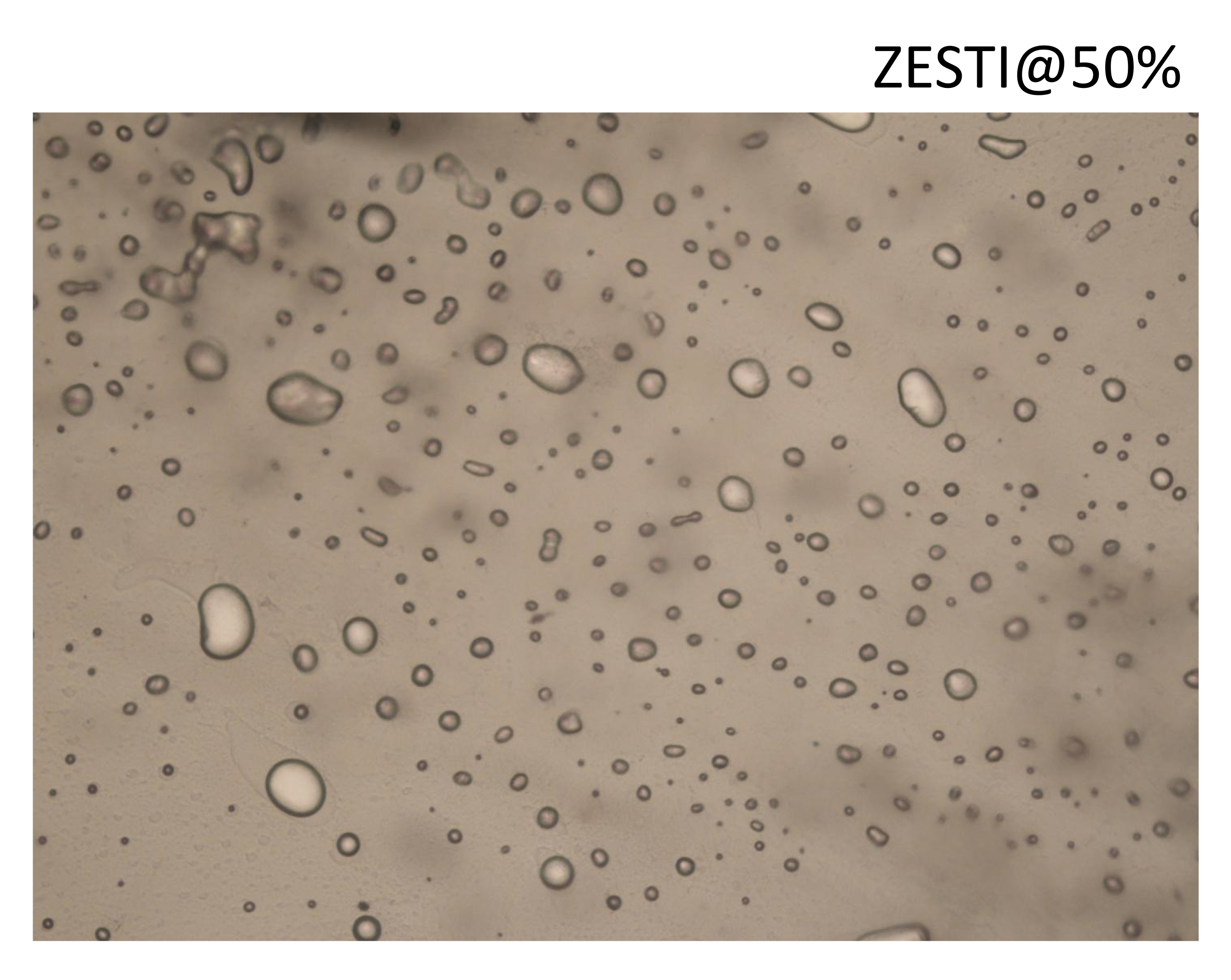


**Fig. S6** Optical microscope image of ZESTI@50%.


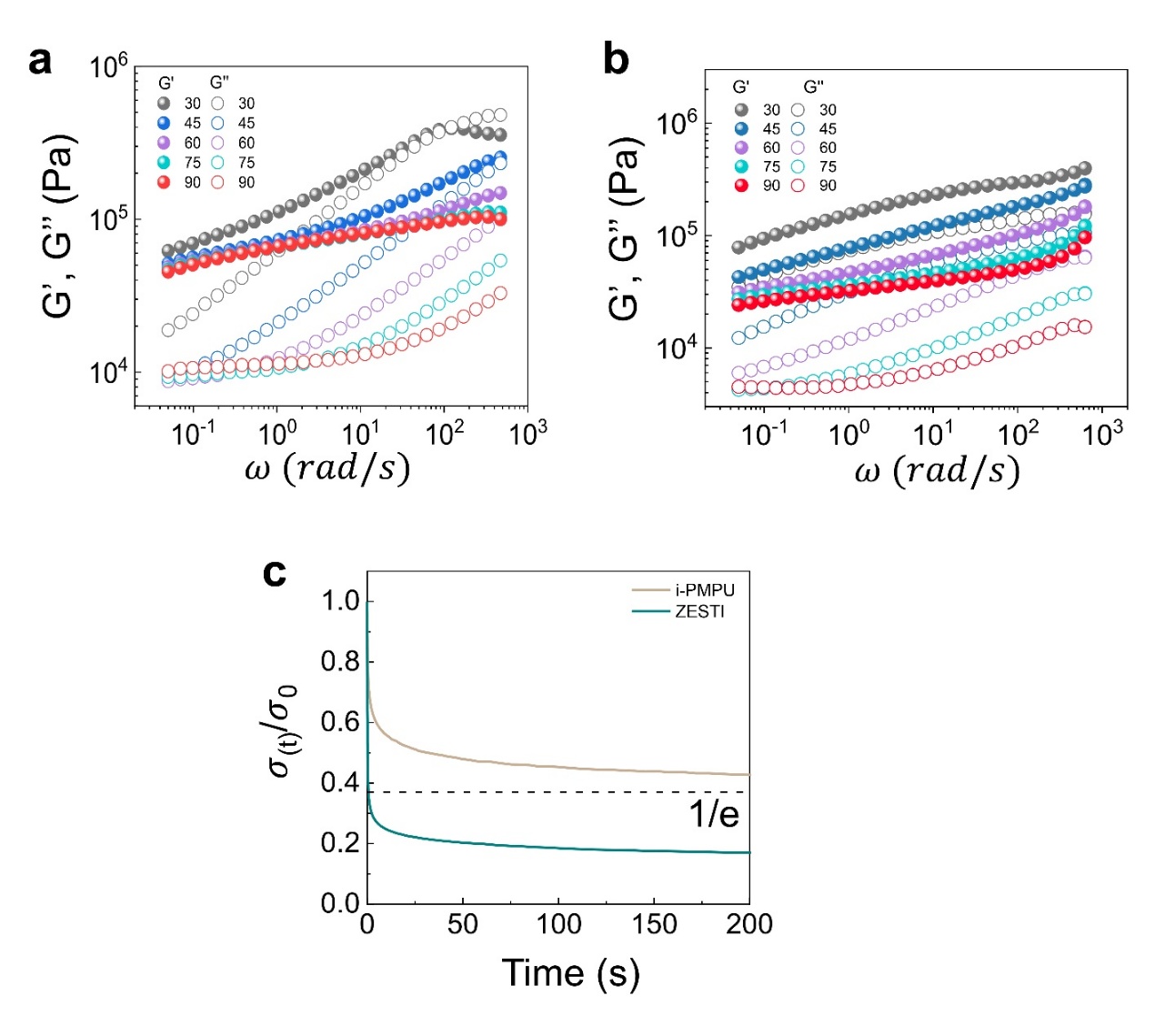


**Fig. S7** Frequency-dependent dynamic storage (filled circle) and loss (open circle) moduli at various temperatures for (a) i-PMPU@30% and (b) ZESTI@30%. (c) Stress relaxation curves of the i-PMPU@30% and ZESTI@30%


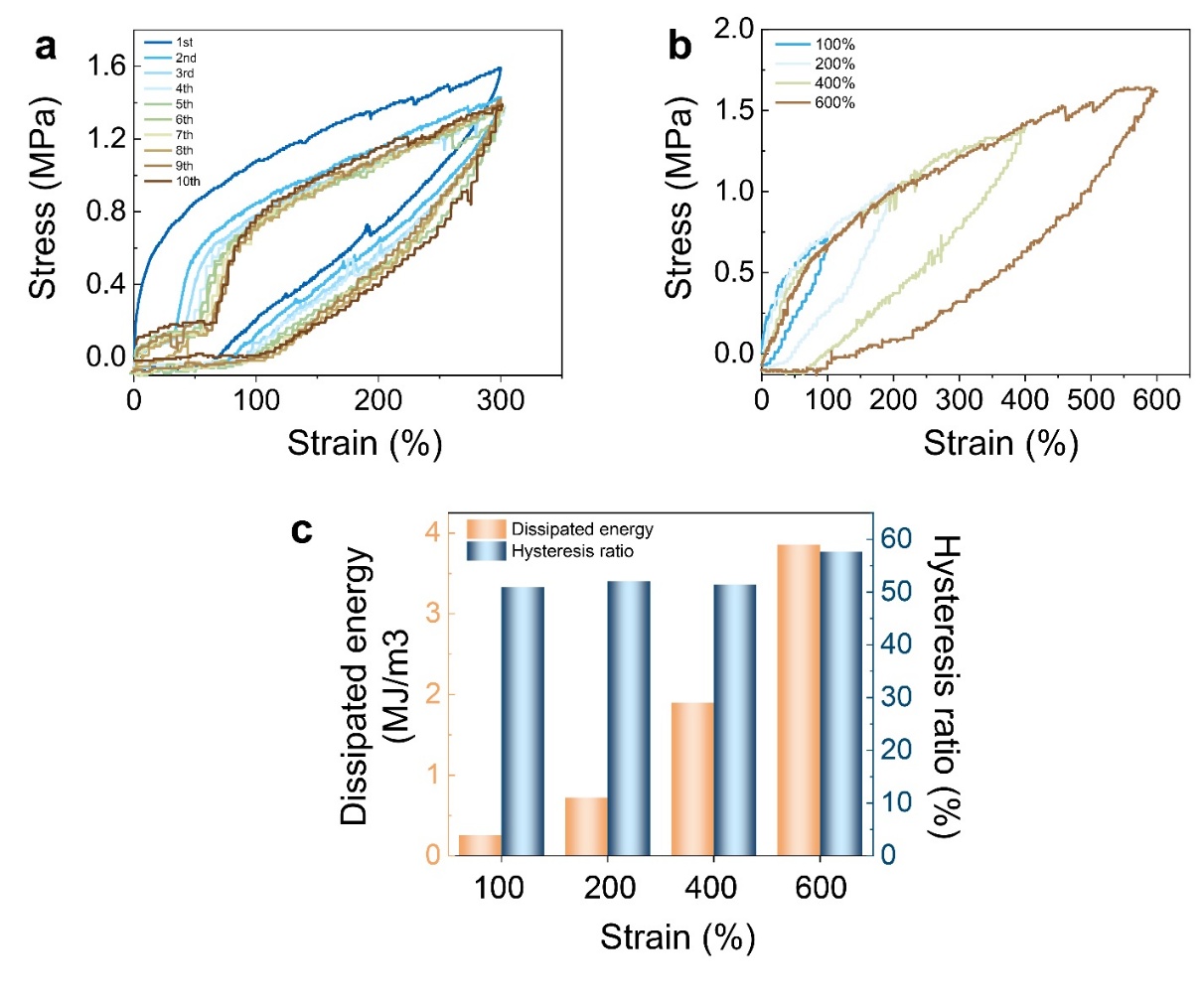


**Fig. S8** Sequential cyclic loading stress-strain curves of ZESTI at (a) 300% strain and (b) maximum strains of 100%, 200%, 400%, and 600%. (c) Corresponding dissipated energy and hysteresis ratio calculated from the hysteresis loops at different maximum strains.


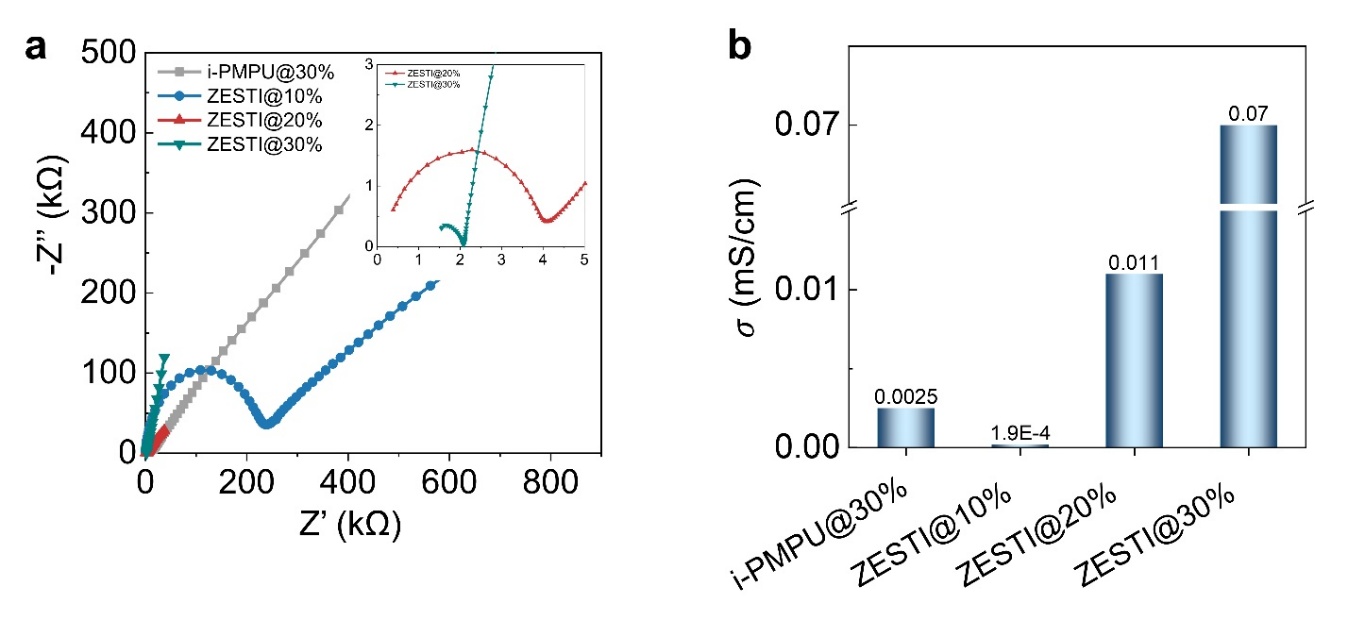


**Fig. S9** (a) Impedance Nyquist plots and (b) Ionic conductivity of ZESTPU with different IL contents.


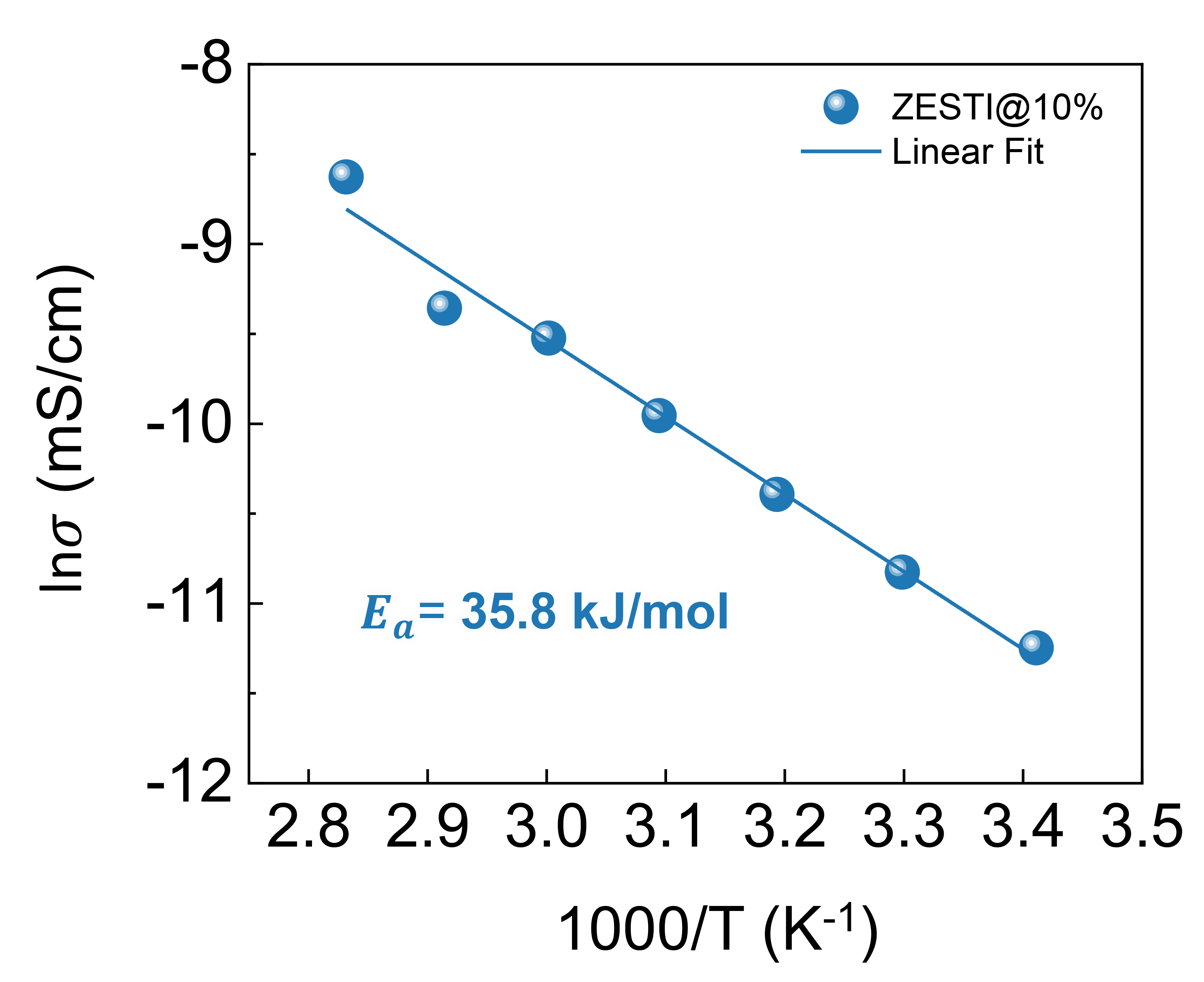


**Fig. S10** Temperature-dependent ionic conductivity analysis of ZESTI@10%.


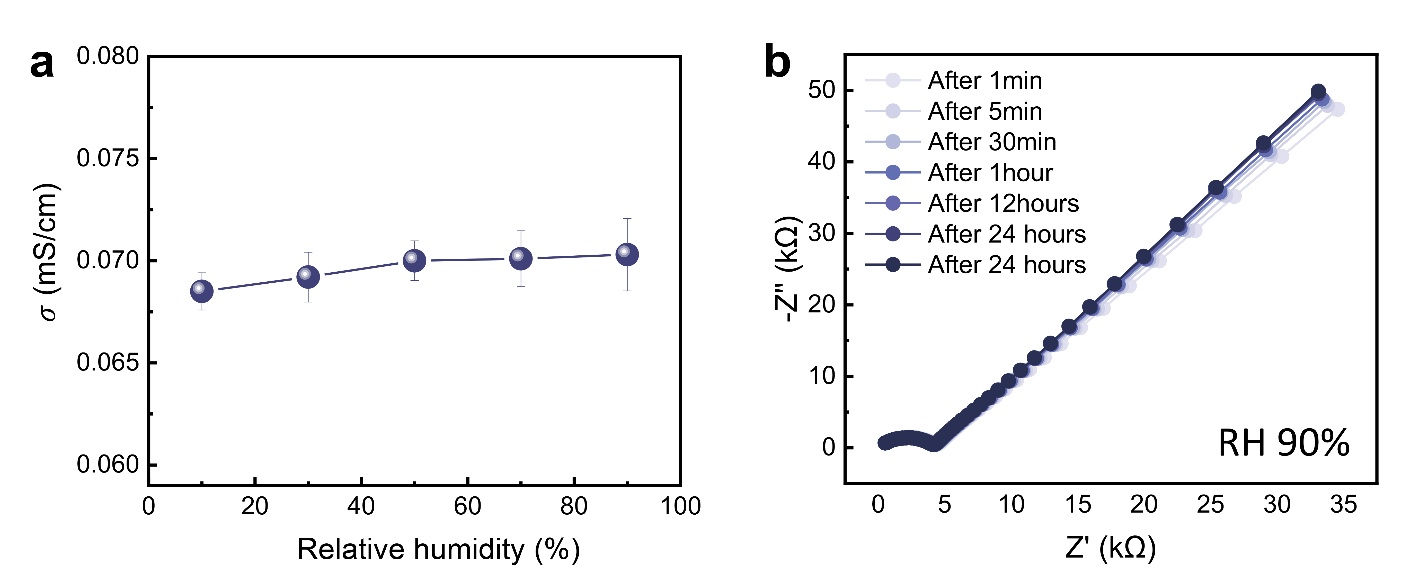


**Fig. S11 Environmental stability of the ionic conductivity of ZESTI.** (a) Ionic conductivity of ZESTI as a function of relative humidity (RH), showing stable conductivity across the measured humidity range. (b) Time-dependent ionic conductivity of ZESTI under continuous exposure to RH 90%.

.


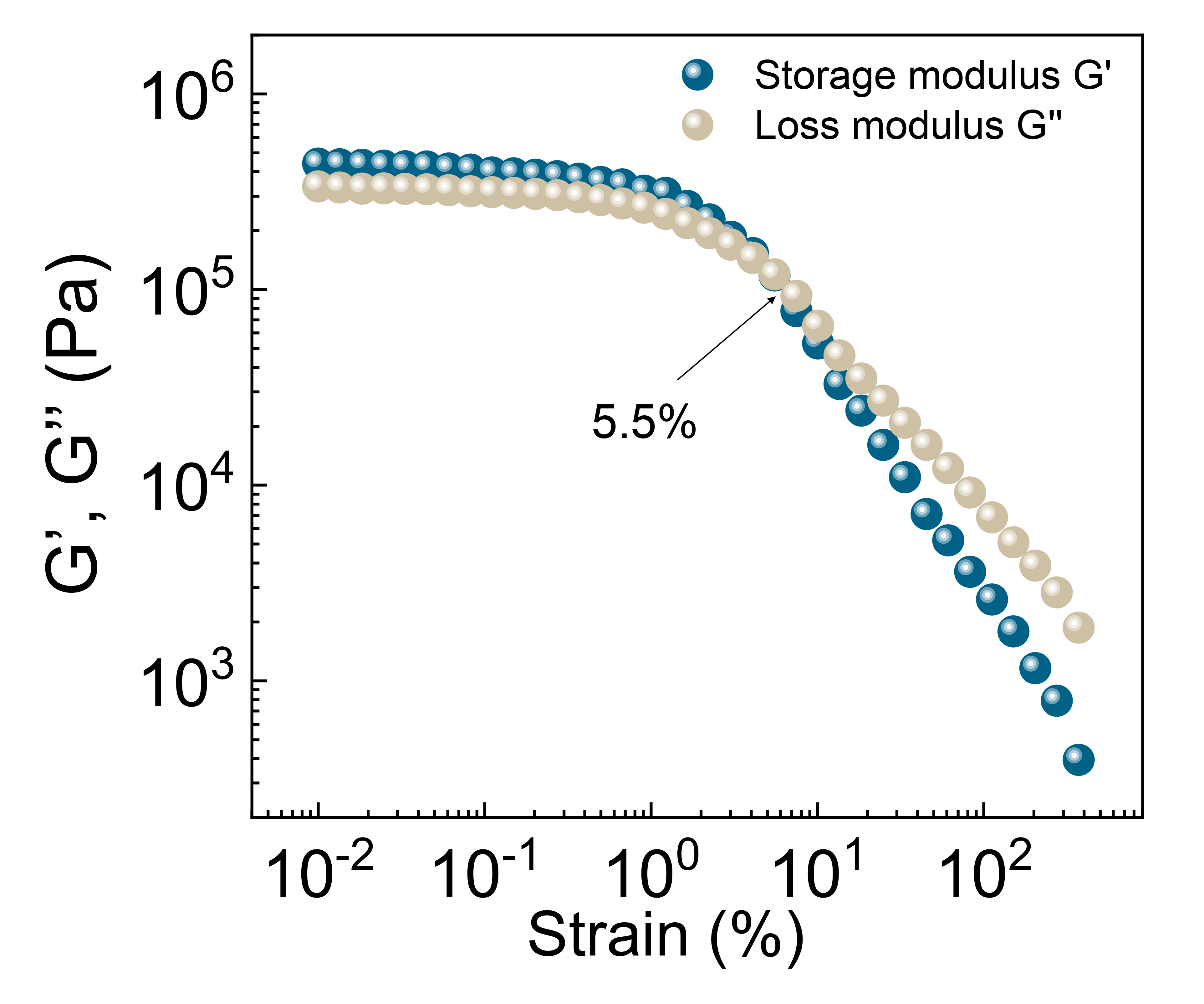


**Fig. S12** Oscillatory strain sweeps of ZESTI@30%.


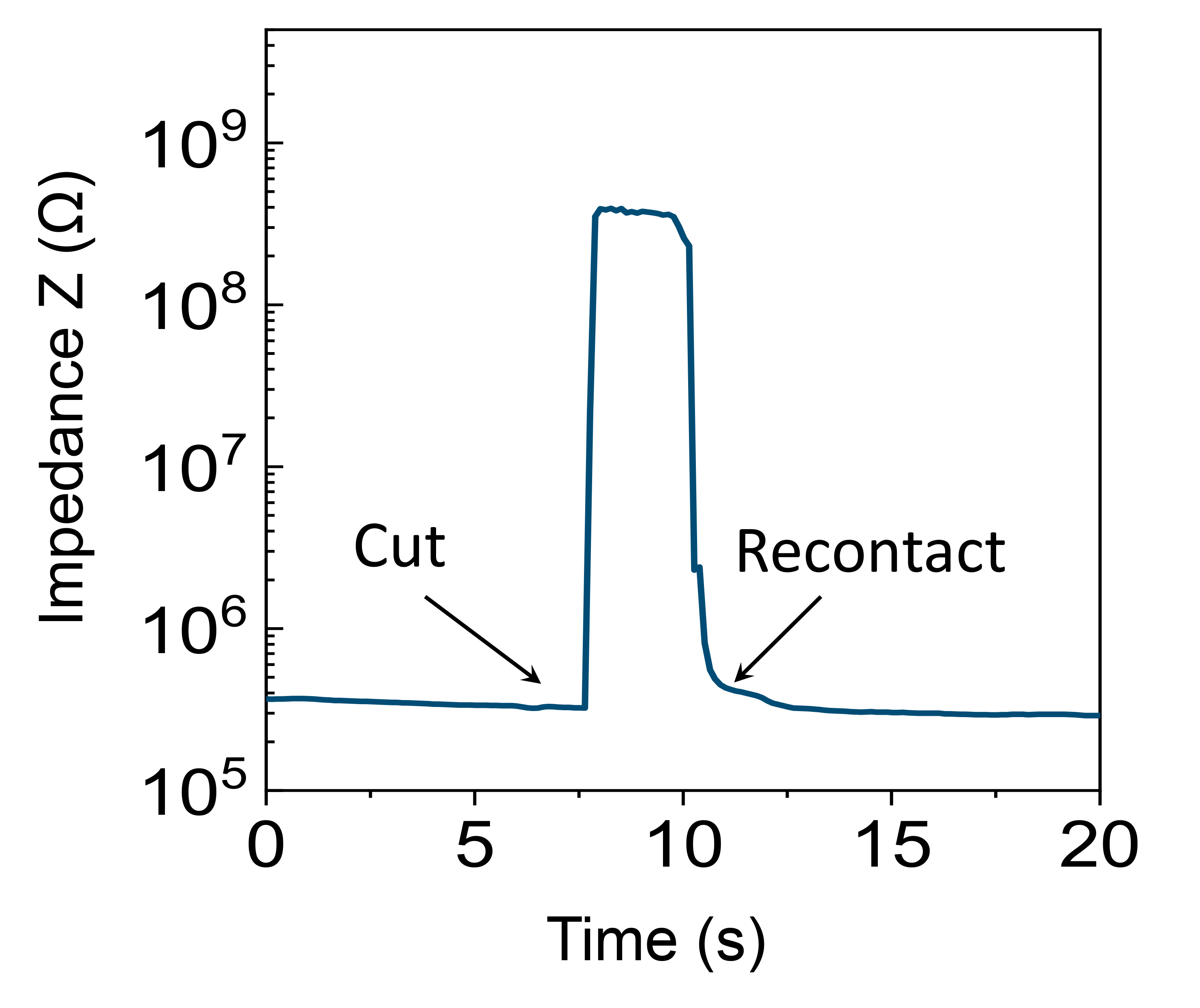


**Fig. S13** Impedance versus test time during the cutting‐healing process for ZESTI@30%.


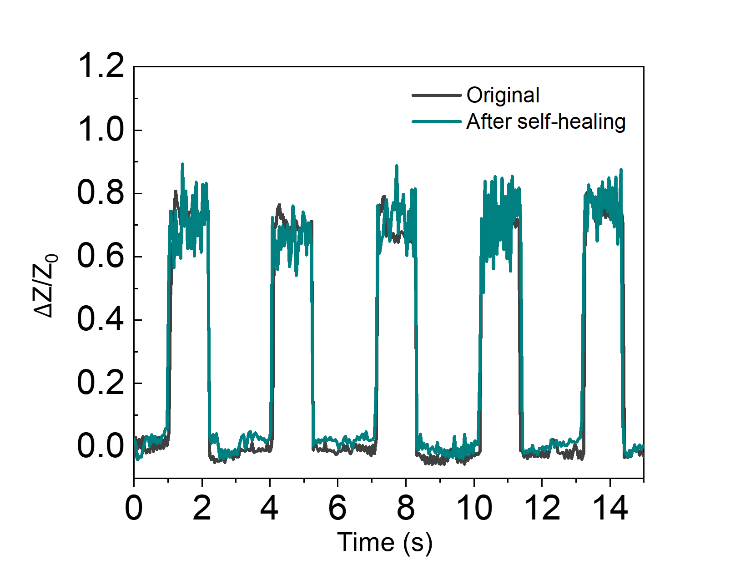


**Fig. S14** Relative impedance changes of the sensing packaging box during packaging of a stainless-steel sphere (Size C) before and after self-healing.


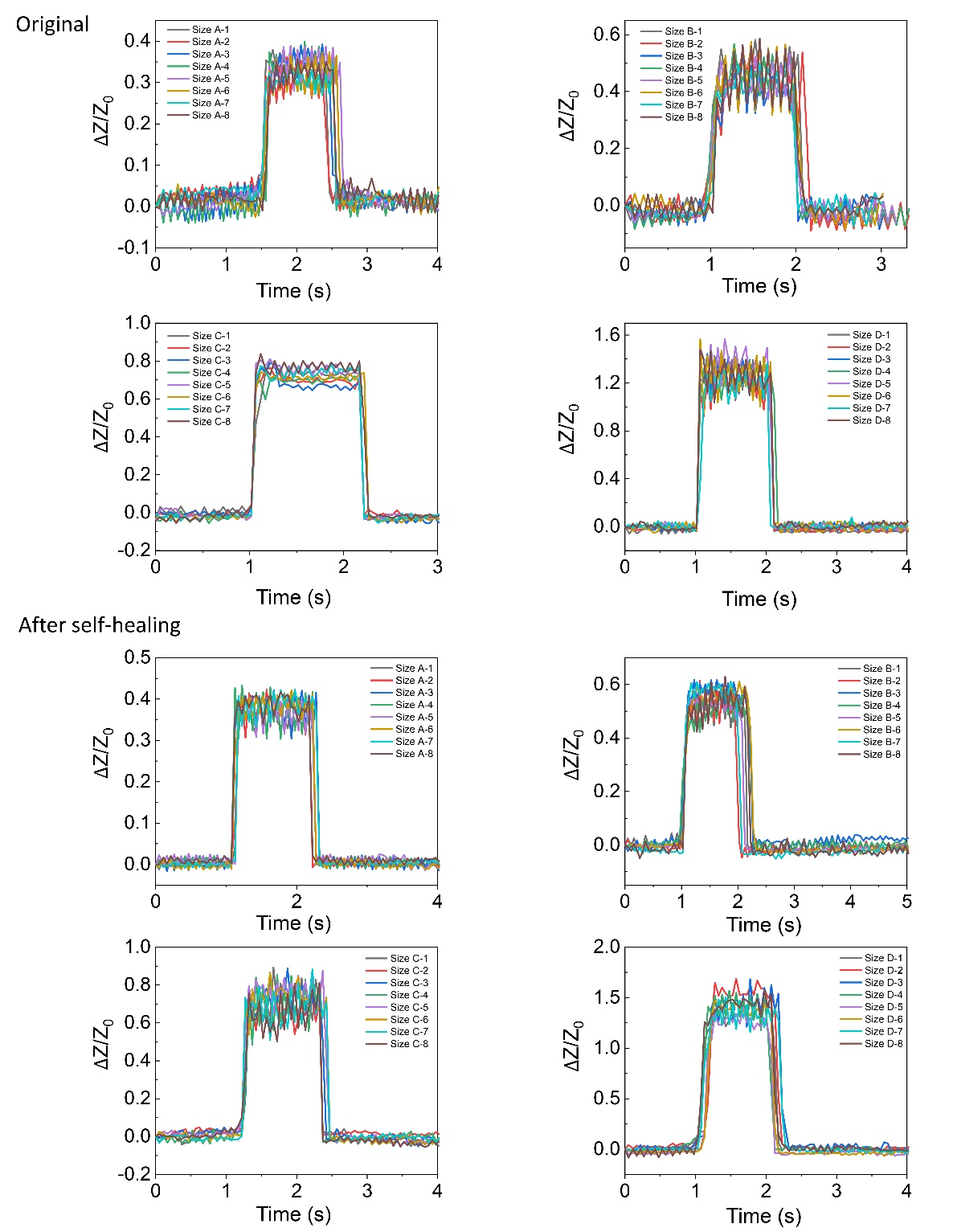


**Fig. S15** Relative impedance changes of the sensing packaging box during packaging of four stainless-steel spheres with different sizes before and after self-healing.


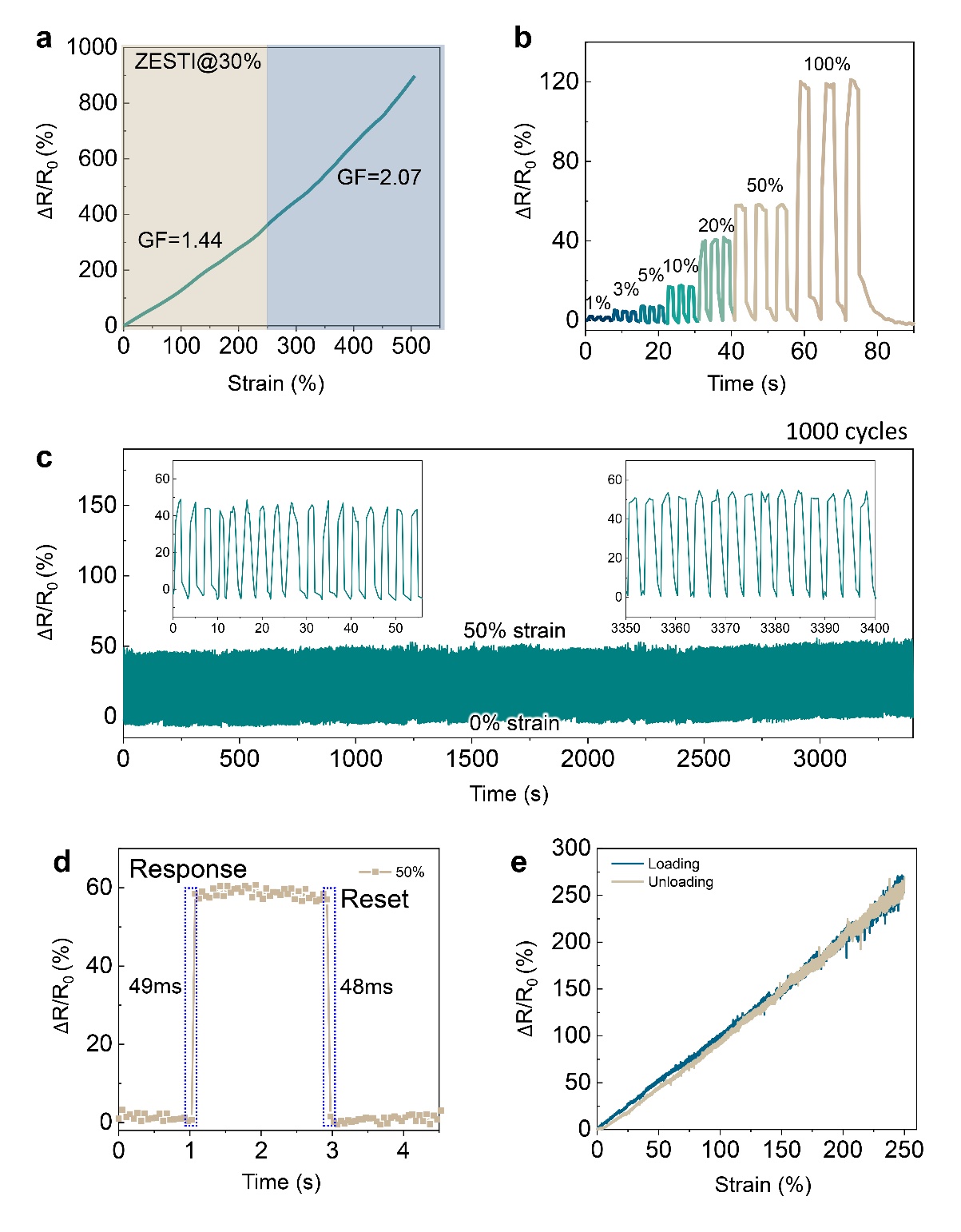


**Fig. S16** (a) Relative resistance change (ΔR/R0) of the ZESTI@30% as a function of strain. (b) Relative resistance response with various cyclic strains. (c) Durability and stability test of the relative resistance change by multicyclic stretching tests with a strain of 50% for 1000 cycles. (d) Transient response time at 50% strain (1V applied bias at 1000 Hz). (e) Relative resistance changes of the loading–unloading cycle within 0–250% tensile strain.

**
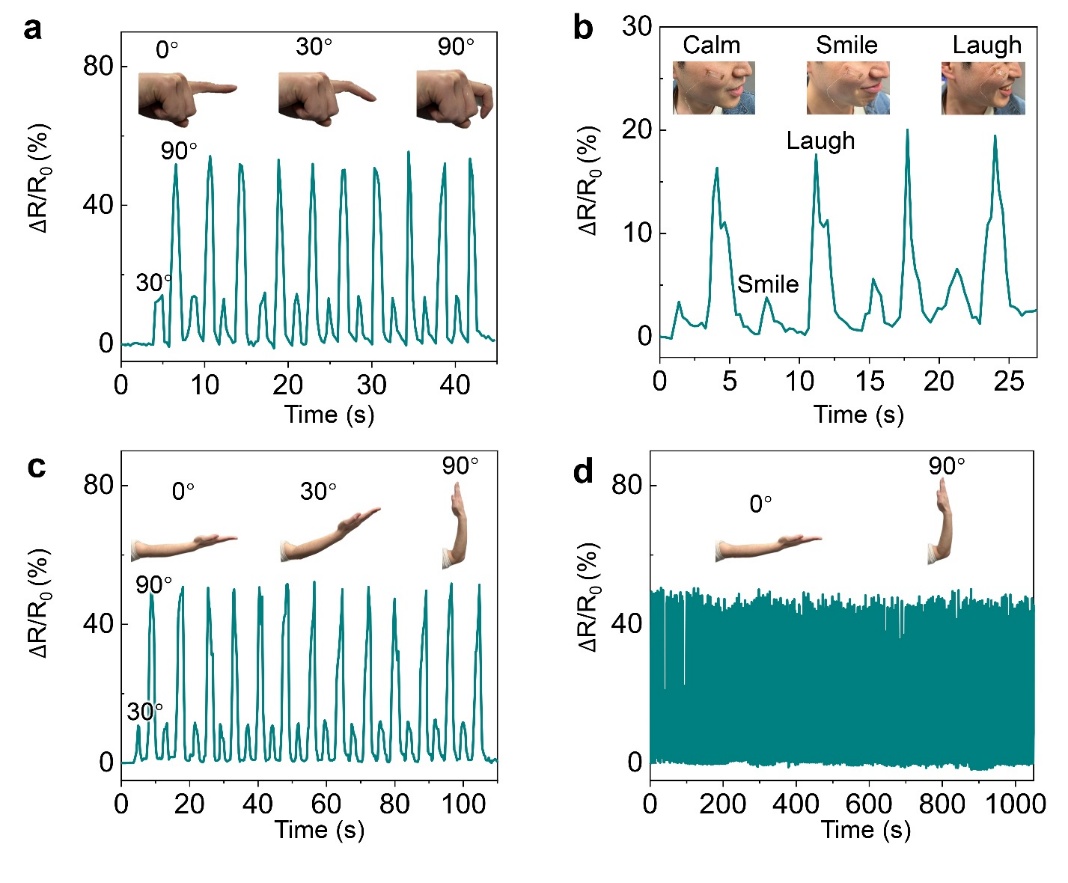
**

**Fig. S17** (a) Relative resistance changes during finger bending (0°-30°-90°). (b) Relative resistance changes during facial expression changes. (c) Relative resistance changes during elbow bending (0°-30°-90°). (d) Relative resistance changes during 1000 seconds of elbow bending (0°-90°).

**Table S1** Compared mechanical property and self-healing capability of self-healable ionogels

| No. | Strain (%) | Stress (MPa) | Modulus (MPa) | Toughness (MJ/m^3^) | Self-healing at RT |
| --- | --- | --- | --- | --- | --- |
| S1 | 1828 | 0.55 | 0.058 | 3.66 | Yes |
| S2 | 916 | 0.59 | 0.8 | 2.33 | Yes |
| S3 | 465 | 1.1 | 1 | 2.7 | No |
| S4 | 600 | 0.12 | 1 | 0.52 | Yes |
| S5 | 1194 | 1.74 | 0.38 | 6.8 | Yes |
| S6 | 504 | 0.225 | 0.18 | 0.5 | Yes |
| S7 | 1500 | 3.2 | 0.1 | 0.49 | Yes |
| S8 | 500 | 3.5 | 1.08 | 5.5 | No |
| S9 | 320 | 6.2 | - | - | No |
| S10 | 404 | 8.94 | - | - | No |
| Our work  (ZESTI@30%) | 1606 | 10.40 | 3.0 | 56.03 | Yes |

**Table S2** Literature references of compared mechanical property and self-healing capability of self-healable ionogels

| **No.** | **Reference** |
| --- | --- |
| S1 | Xu L, Huang Z, Deng Z, et al. A transparent, highly stretchable, solvent‐resistant, recyclable multifunctional ionogel with underwater self‐healing and adhesion for reliable strain sensors[J]. Advanced Materials, 2021, 33(51): 2105306. |
| S2 | Sun L, Huang H, Zhang L, et al. Spider‐silk‐inspired tough, self‐healing, and melt‐spinnable ionogels[J]. Advanced Science, 2024, 11(3): 2305697. |
| S3 | Zeng G, Gao W, Qiu W, et al. Microphase-separation-induced polyzwitterionic ionogel with tough, highly conductive, self-healing and shape–memory properties for wearable electrical devices[J]. Journal of Materials Chemistry A, 2024, 12(44): 30618-30628. |
| S4 | Xia Y, Zhu Y, Zhi X, et al. Transparent self‐healing anti‐freezing ionogel for monolayered triboelectric nanogenerator and electromagnetic energy‐based touch panel[J]. Advanced Materials, 2024, 36(8): 2308424. |
| S5 | Huang Z, Xu L, Liu P, et al. Transparent, mechanically robust, conductive, self-healable, and recyclable ionogels for flexible strain sensors and electroluminescent devices[J]. RSC advances, 2024, 14(38): 28234-28243. |
| S6 | Sun L, Huang H, Ding Q, et al. Highly transparent, stretchable, and self-healable ionogel for multifunctional sensors, triboelectric nanogenerator, and wearable fibrous electronics[J]. Advanced Fiber Materials, 2022: 1-10. |
| S7 | Cao Y, Morrissey T G, Acome E, et al. A transparent, self‐healing, highly stretchable ionic conductor[J]. Advanced Materials, 2017, 29(10): 1605099. |
| S8 | Zhang Z, Qian L, Zhang B, et al. Jellyfish‐inspired polyurea ionogel with mechanical robustness, self‐healing, and fluorescence enabled by hyperbranched cluster aggregates[J]. Angewandte Chemie International Edition, 2024, 63(40): e202410335. |
| S9 | Sun N, Gao X, Wu A, et al. Mechanically strong ionogels formed by immobilizing ionic liquid in polyzwitterion networks[J]. Journal of Molecular Liquids, 2017, 248: 759-766. |
| S10 | Xie J, Li X, Liu J, et al. A transparent and robust ionogel prepared via phase separation for sensitive strain sensing[J]. Journal of Materials Chemistry A, 2024, 12(26): 16160-16173. |

**Table S3** Elemental analysis results of ZESTPU

| Sample | Elemental content obtained by EA (%) | | | |
| --- | --- | --- | --- | --- |
|  | **Nitrogen** | **Carbon** | **Hydrogen** | **Sulphur** |
| ZESTPU | 4.70 | 60.24 | 9.92 | 1.64 |

**Table S4** Average phase distance obtained from SAXS analysis

|  | $\boldsymbol{q}\boldsymbol{max}$ (nm^-1^) | Average phase distance (nm) |
| --- | --- | --- |
| PMPU | 0.50 | 12.56 |
| ZESTPU | 0.38 | 16.52 |
| ZESTI@10% | 0.40 | 15.70 |
| ZESTI@30% | 0.45 | 13.95 |

**Table S5** Comparison of ZESTI with representative zwitterionic ionogels

|  | Conventional zwitterionic ionogels | ZESTI |
| --- | --- | --- |
| Polymer  architecture | fully zwitterionic network or zwitterionic copolymer scaffold | zwitterionic side chains grafted onto microphase-separated polyurethane |
| Role of  zwitterion | ion coordination, ion dissociation, self-healing sites | hard-domain reinforcement, IL localization, healing, ion hopping |
| Microphase  regulation | usually not the central design target | selective IL localization preserves PU hard-segment aggregation |
| Dynamic  interaction | ion–dipole or dipole–dipole interaction | coupled dipole–dipole and ion–dipole interactions |
| Self-healing | reported in selected systems | autonomous healing under ambient conditions with mechanical and electrical recovery |
| Main novelty | ionic gel electrolyte or self-healable ionogel | simultaneous microphase regulation, mechanical reinforcement, self-healing and ion transport |

**Table S6** Mechanical properties of different PU and ionogels

|  | Strain (%) | Tensile strength (MPa) | Young’s modulus (MPa) | | Toughness (MJ/m^3^) |
| --- | --- | --- | --- | --- | --- |
| PMPU | 1296 | 22.7 | 30.5 | 127.2 | |
| i-PMPU@30% | 763 | 6.24 | 21.3 | 30.7 | |
| ZESTPU | 837 | 45.5 | 291 | 216.0 | |
| ZESTI@30% | 1606 | 10.40 | 3.0 | 56.03 | |

**Table S7** Mechanical properties of ZESTPU with different IL contents

|  | Strain (%) | Tensile strength (MPa) | Young’s modulus (MPa) | Toughness (MJ/m^3^) | |
| --- | --- | --- | --- | --- | --- |
| ZESTPU | 837 | 45.5 | 291 | | 216.0 |
| ZESTI@10% | 1481 | 11.0 | 18.3 | | 74.91 |
| ZESTI@30% | 1606 | 10.40 | 3.0 | | 56.03 |
| ZESTI@50% | 2266 | 3.17 | 1.1 | | 35.66 |

**Table S8** Dissipated energy and hysteresis ratio of ZESTI at different strain

| Strain | U_loading_ (MJ/m^3^) | U_unloading_ (MJ/m^3^) | Dissipated energy (MJ/m^3^) | | Hysteresis ratio (%) |
| --- | --- | --- | --- | --- | --- |
| 100% | 0.499 | 0.245 | 0.254 | 50.9 | |
| 200% | 1.386 | 0.665 | 0.721 | 52.02 | |
| 400% | 3.692 | 1.794 | 1.898 | 51.4 | |
| 600% | 6.688 | 2.830 | 3.858 | 57.7 | |
